# Elucidating a molecular mechanism that the deterioration of porcine meat quality responds to increased cortisol based on transcriptome sequencing

**DOI:** 10.1038/srep36589

**Published:** 2016-11-11

**Authors:** Xuebin Wan, Dan Wang, Qi Xiong, Hong Xiang, Huanan Li, Hongshuai Wang, Zezhang Liu, Hongdan Niu, Jian Peng, Siwen Jiang, Jin Chai

**Affiliations:** 1 Agricultural Ministry Key Laboratory of Swine Breeding and Genetics & Key Laboratory of Agricultural Animal Genetics, Breeding, and Reproduction of Ministry of Education, Huazhong Agricultural University, Wuhan, China; 2 The Cooperative Innovation Center for Sustainable Pig Production, Wuhan, China; 3 Hubei Key Laboratory of Animal Embryo Engineering and Molecular Breeding, Institute of Animal Husbandry and Veterinary, Hubei Academy of Agricultural Science, Wuhan, China; 4 Department of Animal Nutrition, Huazhong Agricultural University, Wuhan, China

## Abstract

Stress response is tightly linked to meat quality. The current understanding of the intrinsic mechanism of meat deterioration under stress is limited. Here, male piglets were randomly assigned to cortisol and control groups. Our results showed that when serum cortisol level was significantly increased, the meat color at 1 h postmortem, muscle bundle ratio, apoptosis rate, and gene expression levels of calcium channel and cell apoptosis including *SERCA1*, *IP3R1*, *BAX*, *Bcl-2*, and *Caspase-3*, were notably increased. However, the value of drip loss at 24 h postmortem and serum CK were significantly decreased. Additionally, a large number of differentially expressed genes (DEGs) in GC regulation mechanism were screened out using transcriptome sequencing technology. A total of 223 DEGs were found, including 80 up-regulated genes and 143 down-regulated genes. A total of 204 genes were enriched in GO terms, and 140 genes annotated into in KEGG database. Numerous genes were primarily involved in defense, inflammatory and wound responses. This study not only identifies important genes and signalling pathways that may affect the meat quality but also offers a reference for breeding and feeding management to provide consumers with better quality pork products.

Muscle is an important part of the carcass composition and is usually the main source of meat products for humans. With improvements in living standards and consumption levels, people have higher demands for meat quality of animal products. In livestock production, the process of transport and slaughter causes a variety of stresses, which affects the meat quality and influences the performance of the pig products^1^. Meat quality is affected by the physiological status of pre-slaughter, and poor meat quality usually results from released cortisol^2^. In general, plasma concentrations of cortisol are reported to change when animals are subjected to stressors^3^. Previous studies have shown that pre-slaughter factors, such as the feeding system and fasting period, also negatively affect meat quality by increasing serum cortisol levels^4^. In addition, other studies suggested that when pigs were transported during the cold season, there were higher levels of cortisol and a greater quantity of tough meat^5^. Stress can lead to an increase in stress hormones, such as ACTH and corticosteroid^6^, which is likely the important factor that affects meat quality.

As a glucocorticoid (GC), cortisol is a main stress hormone that is secreted by the adrenal cortex. It is governed by the hypothalamic-pituitary-adrenal (HPA) axis in the stress state^7^, and the concentration of cortisol in plasma is often used as a reliable indicator of the degree of stress^8^. In addition, cortisol as a primary stressor also affects muscle proteolysis and meat quality in piglets^9^. Different concentrations of cortisol in pig muscle had effects on meat qualities such as color, drip loss, pH^10^.

Several studies focusing on meat quality traits reported that some proteins including ATF4, IGF-1, CAST and MoyD1 reflected the meat quality parameters (pH, conductivity, meat color, shear force, and drip loss)^11^. Other studies indicate that the change of Desmin depends on pH and that Desmin has effects on muscle fibre composition^12^. In addition, calpain1 and calpain2 are main factors that cause muscle fiber protein degradation directly related to the degree of meat tenderness^13^. These proteins were selected based on their function regarding muscle development and metabolism and their association with meat quality traits under cortisol treatment.

High levels of cortisol (GC) resulting from stress may play a major role in reducing meat quality. Reports indicated that *IP3R1* and *SERCA1* induced by GC cause a calcium imbalance that stimulates cell apoptosis. In addition, *BAX*, *Bcl-2*, and *Caspase-3* induced by GC may change the mitochondrial membrane permeability in C2C12 myoblasts^14^. Calcium regulation genes such as *αRYR*, *βRYR*, *CASQ1*, and *SERCA1* in heat-stressed turkey breast muscle are associated with the degree of meat quality^15^. Another study reported that apoptosis factors and caspase activation pathways can affect beef muscle^16^. However, these data are not sufficient to elucidate the intrinsic mechanism of the effect of GC on the meat quality deterioration, and there are few studies on the internal control genes and related pathways. Therefore, we selected a high-throughput sequencing platform to screen out genes and pathways.

Transcriptome sequencing is widely used in basic research, clinical diagnosis and drug development. In general, the transcriptome refers to the sum of all RNAs of a cell, a tissue or an organism in a specific environment or physiological condition^17^. A large number of candidate genes between the psoas and the *longissimus dorsi* (LD) were obtained by cDNA microarray chip of porcine skeletal muscle^18^. A total of 315 differentially expressed genes (DEGs), including 240 genes annotated to the DAVID database, were obtained by comparing the LD sequencing results of the Chinese pig and imported pig^19^. In our research group, a significant number of DEGs associated with labor onset have been obtained via comparison of sequencing data from a shorter gestation period and a normal gestation period^20^. Therefore, transcriptome sequencing is an effective method for obtaining DEGs regarding important pig economical traits. Further functional annotation and enrichment analysis of DEGs or transcripts provided the molecular basis for the following biological research^21^. This study screened DEGs by transcriptome sequencing, performed functional annotation and signalling pathway analysis of DEGs, and explored molecular regulation mechanism of GC at the transcript level.

This study investigates the intrinsic molecular mechanism of meat deterioration under stress by evaluating the LD, including such parameters as the blood and meat quality index, muscle bundle rate, and apoptosis rate and also by obtaining DEGs via high-throughput transcriptome sequencing technology. The objective of this study is to provide a new perspective for improving meat quality and animal welfare.

## Results

### Meat color, conductivity, pH, and drip loss

**Figure 1:**
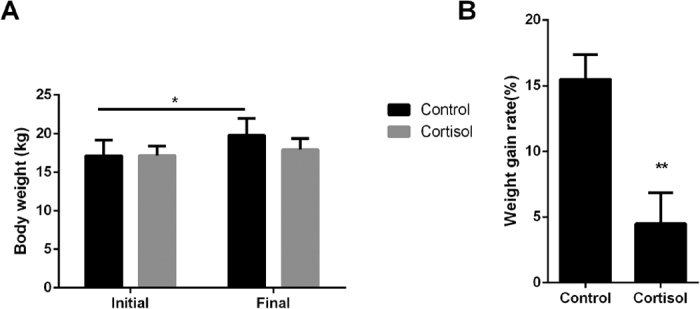
Effects of cortisol on pig weight. (**A**) Body weight. (**B**) Weight gain rate. The experimental group received a commercial diet with the addition of cortisol (120 mg/kg diet) for 7 days. The control group received a commercial diet (*P < 0.05, **P < 0.01).

**Figure 2:**
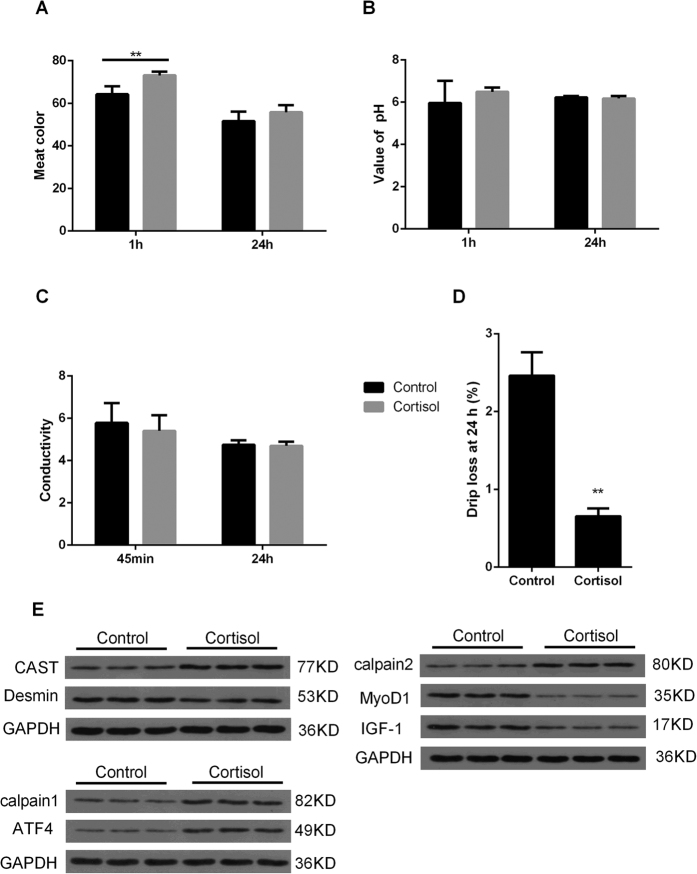
Effect of cortisol on meat quality traits and protein related to meat quality. (**A**) Meat color. (**B**) pH. (**C**) Conductivity. (**D**)Drip loss. (**E**) Western blot analysis of proteins related to meat quality trait. All values are the means ± standard deviations (SDs). Different superscript letters indicate significant among-group differences.

To examine the effect of stress hormones on meat quality, all piglets were slaughtered at 42 days of age. In the control group, the final body weight (Day 42) was significantly higher than the initial weight (Day 35) (*P* < 0.05) (Fig. 1A). Additionally, the weight gain rate of the cortisol group was significantly lower than the control group (Fig. 1B). The meat color value at 1 h postmortem was significantly increased (*P* < 0.01), and there was no significant difference in color at 24 h postmortem between the cortisol and control groups, but the color value showed an increasing trend (Fig. 2A). In this experiment, there was no difference in pH and conductivity at 45 min and 24 h postmortem between the two groups (Fig. 2B,C). However, drip loss in the cortisol group was significantly lower (*P* < 0.01) than the control group (Fig. 2D). Taken together, these results suggest that higher meat color at 1 h and lower drip loss may correlate with the accelerated degradation of skeletal muscle but not with the effects of pH and conductivity.

### Western blot analysis of proteins related to meat quality trait

To determine whether cortisol causes a decline in meat quality, several proteins associated with meat quality traits were detected. Western blot analysis demonstrated lower protein levels of Desmin, IGF-1, and MyoD1 in the cortisol group compared to the control group. The protein levels of ATF4, CAST, calpain1, and calpain2 were higher in the cortisol group than the control group (Fig. 2E and Supplementary Fig. S1).

### Blood index examinations

**Figure 3:**
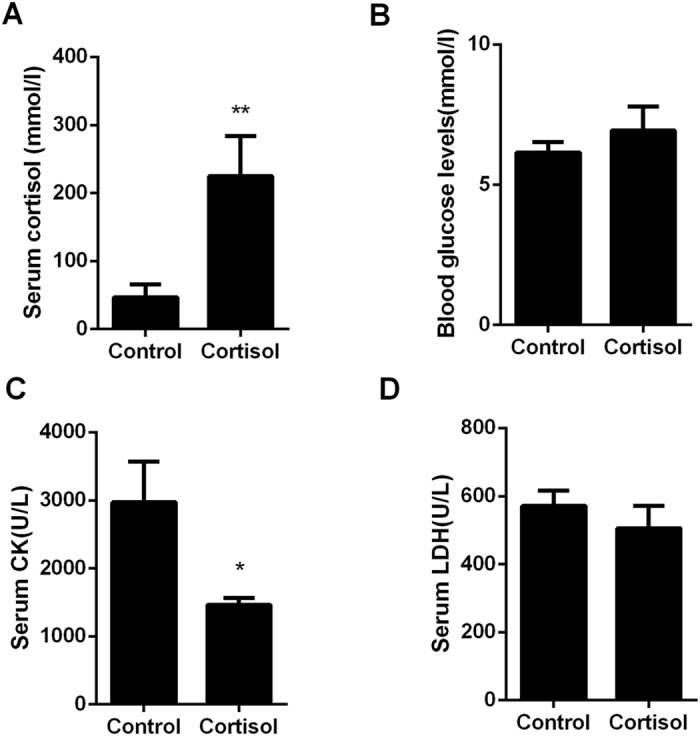
Blood biochemical index of tissue. (**A**) Cortisol. (**B**) Blood glucose. (**C**) CK. (**D**) LDH.

To further characterize the effect of cortisol on blood parameters, such as blood glucose, creatine kinase (CK) and lactate dehydrogenase (LDH), these blood parameters were measured. The serum cortisol concentrations in the cortisol group were significantly higher than the control group (*P* < 0.01) (Fig. 3A), and the CK levels in the cortisol group were significantly lower (*P* < 0.05) than the control group (Fig. 3C). However, the blood glucose and LDH levels (Fig. 3B,D) in the cortisol group were not affected.

### Histological examination and apoptosis analysis

**Figure 4:**
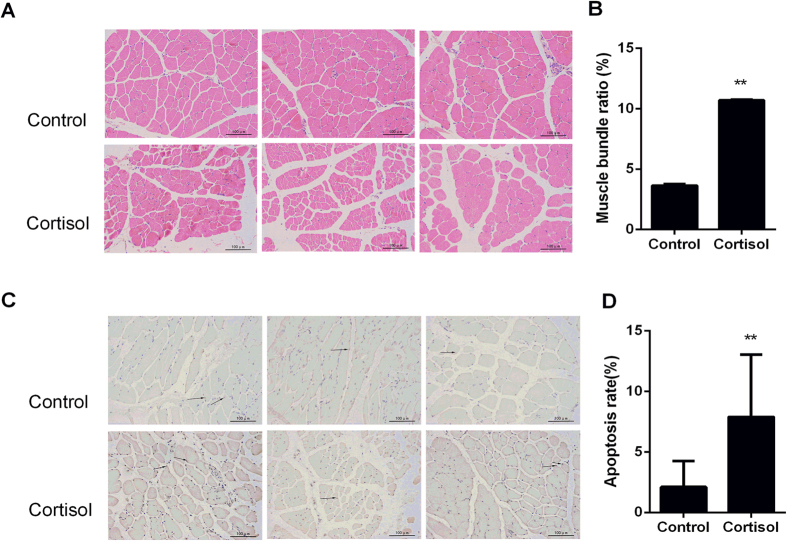
Tissue damage caused by cortisol. (**A**) HE. (**B**) Muscle bundle ratio (%). (**C**) TUNEL. (**D**) Apoptosis ratio. The cortisol group significantly differed from the control group (*P* < 0.01).

In order to quantify the induction of apoptosis by cortisol, the degree of muscle injury and the number of apoptotic cells were measured. Haematoxylin-eosin (HE) staining revealed severe tissue injuries in the cortisol group (Fig. 4A), and the cortisol group exhibited a much higher muscle bundle rate than the control group (*P* < 0.01) (Fig. 4B). Compared with the control group, the number of apoptotic cells greatly increased (Fig. 4C), and the apoptosis rate was significantly increased (*P* < 0.01) (Fig. 4D). These results demonstrate that cortisol changes the physical and chemical properties of muscle by inducing cell apoptosis.

### Calcium-channel-related and apoptosis-related genes

**Figure 5:**
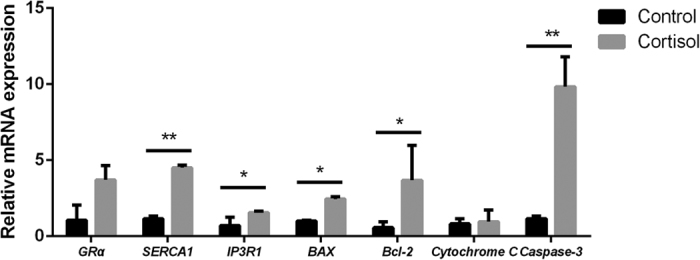
Quantitative RT-PCR validation of calcium-channel-related and apoptosis-related genes. The expression levels of *IP3R1*, *BAX* and *Bcl-2* were significantly higher (*P* < 0.05) in cortisol-treated group than the control group. *SERCA1* and *Caspase-3* were significantly increased (*P* < 0.01) in the group fed cortisol.

To determine whether the stress hormone-induced meat quality decline is associated with calcium-channel-related and apoptosis-related genes, we measured the expression of these genes using qRT-PCR technology. Compared with the control group, the cortisol group had significantly higher expression levels of calcium-channel-related genes (*SRECA1* and *IP3R1*) and apoptosis-related genes (*BAX*, *Bcl-2* and *Caspase-3*) (*P* < 0.05). However, the expression of *Cytochrome C* and *GRα* in the cortisol group were not affected but exhibited an increasing trend (Fig. 5).

### Sequence and assembly

In total, 28.96 Gb clean data were obtained from 6 samples of the RNA-seq, and the clean data from each sample reached 4.37 Gb. The percentage of base Q30 was greater than 87.10%. Then, clean reads were aligned with those of the specified reference genome, and the alignment efficiency ranged from 72.20% to 73.30% (Supplementary Table S1). A total of 1408 new genes were discovered, and 1178 of them were annotated. DEGs were identified based on the gene expression of different samples. Their functional annotation and enrichment analysis were then performed.

### Identification and functional analysis of DEGs

**Figure 6:**
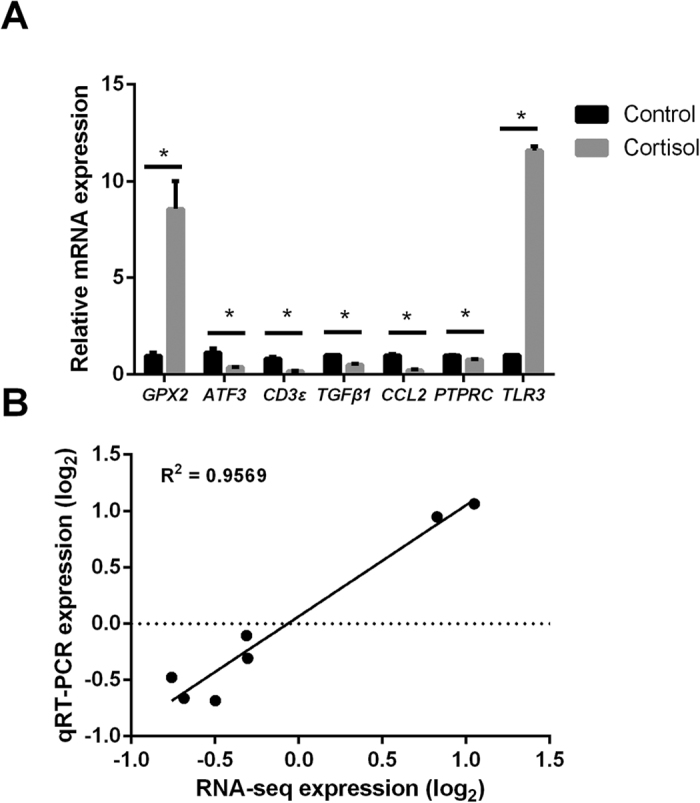
Quantitative RT-PCR validation of differentially expressed genes. (**A**) The qRT-PCR-based validation of *GPX2*, *ATF3*, *CD3ε*, *TGFβ1*, *CCL2*, *PTPRC* and *TLR3* expression levels in muscle. (**B**) Correlation of fold change values measured using q-PCR and RNA-Seq methods.

To validate the reliability of the mRNA sequencing results, qRT-PCR was performed to detect the levels of 7 randomly selected differential mRNAs from the differential expression data (Fig. 6A). The qRT-PCR results showed that most of the mRNAs were consistent with the mRNAs profiling results. To confirm the gene expression pattern, 7 genes including *GPX2*, *ATF3*, *CD3ε*, *TGFβ1*, *TLR3*, *PTPRC*, and *CCL2* were selected for evaluation by qRT-PCR. The results showed that these genes were consistent with those of the RNA sequencing results (Pearson correlation coefficient = 0.9569) (Fig. 6B).

**Figure 7:**
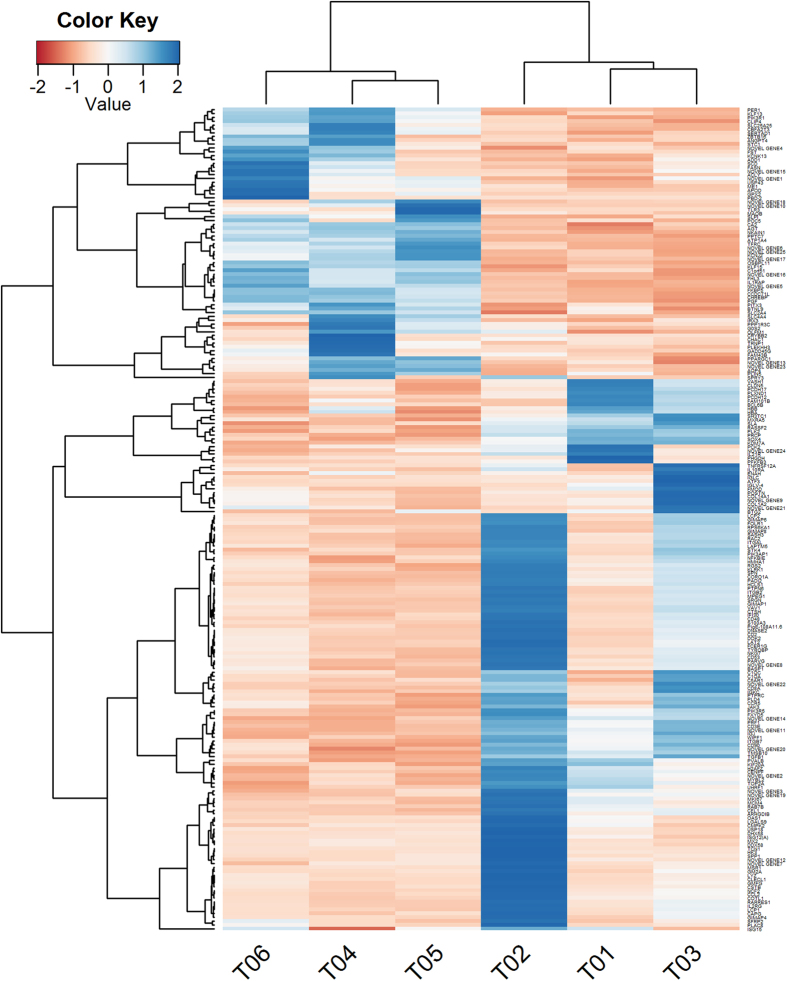
Heat map of DEGs in *longissimus dorsi* muscle. Each row of colored tiles in the grid corresponds to one gene, and each column represents a sample. The gene tree drawn to the right of the heat map displays the clustering of genes.

A total of 223 DEGs were screened, containing 80 up-regulated genes and 143 down-regulated genes (Supplementary Table S2). Of these DEGs, 216 were annotated to the DAVID database, 204 were annotated to the GO database, and 140 were annotated to the KEGG functional pathway. The correlation analysis of the two samples was performed after high-throughput sequencing data were obtained to examine the correlation of expression of the same gene from different biological samples. The test results showed that the correlation between two treatment groups was very high and that R^2^ was greater than 0.92 (Supplementary Fig. S2). A total of 223 DEGs from six sequencing samples was divided into 2 categories. The results showed that T01, T02, and T03 clustered into one category and that T04, T05, and T06 clustered into the other category (Fig. 7). A total of 80 DEGs was highly expressed in the cortisol group (T04, T05, T06), whereas 143 DEGs were highly expressed in the control group (T01, T02, T03). Hierarchical clustering revealed perfect separation between the muscle tissues in the cortisol and control groups that may be indicative of divergences in the stressed state.

### Immunohistochemistry and western blot analysis of TLR3 and IL-1RAP

**Figure 8:**
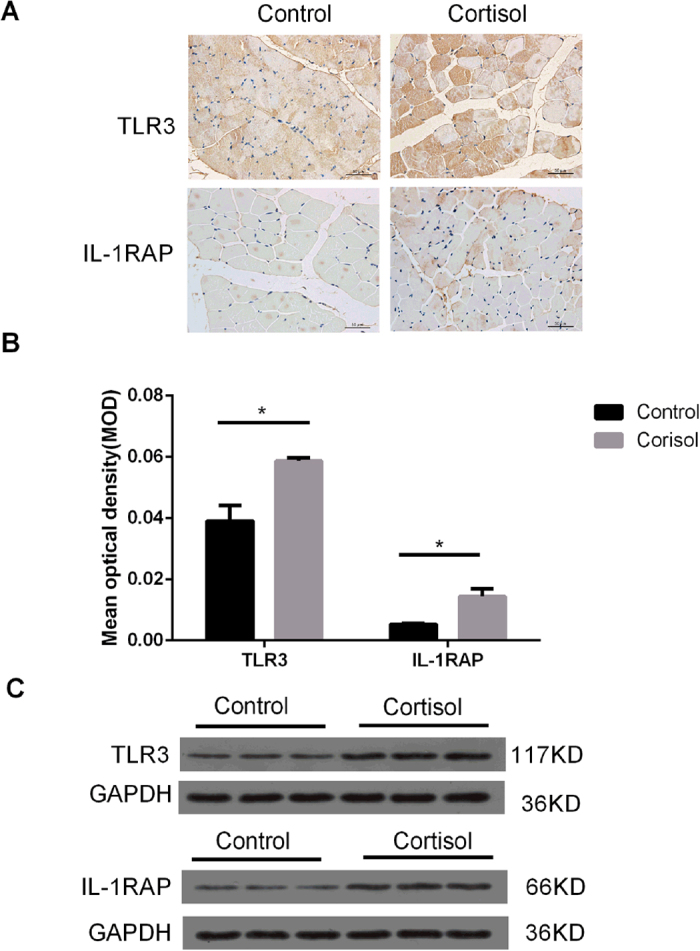
Identification of TLR3 and IL-1RAP. (**A**) Immunohistochemistry. (**B**) Mean optical density (MOD), integrated optical density (IOD), MOD = IOD/area (sum). The IOD of TLR3 and IL-1RAP expression and sum area were calculated using Image-Pro Plus software. (**C**) Western blot.

*TLR3* and *IL-1RAP* were up-regulated in the cortisol group, and their enrichment score were high, which may play an important role in the mechanism of meat quality decline. Statistical data revealed that the mean optical density (MOD) of TLR3 and IL-1RAP significantly increased in the cortisol group compared to the control group based on immunohistochemistry analysis (*P* < 0.05) (Fig. 8A,B). The data of integrated optical density (IOD) and sum area were calculated using Image-Pro Plus software. MOD is the ratio of IOD to the sum area. In addition, the expression levels of TLR3 and IL-1RAP of the cortisol group were higher than that of control group, which were confirmed by western blot analysis (Fig. 8C and Supplementary Fig. S1).

### Kyoto encyclopaedia of genes and genomes (KEGG) pathway and gene ontology (GO) term analyses

**Figure 9:**
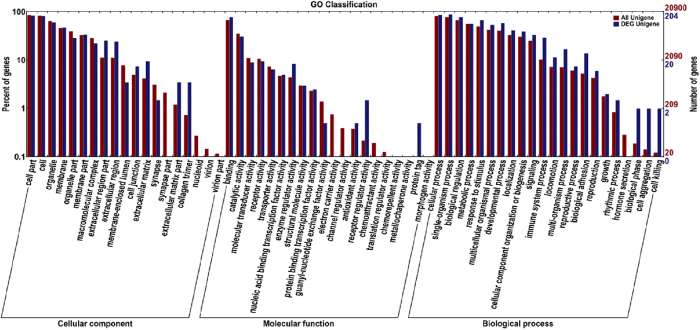
Significantly enriched Gene Ontology (GO) terms of DEGs. The horizontal coordinate is the GO classification. The left side of the vertical coordinate is the percentage of genes and the right is the number of genes.

**Figure 10:**
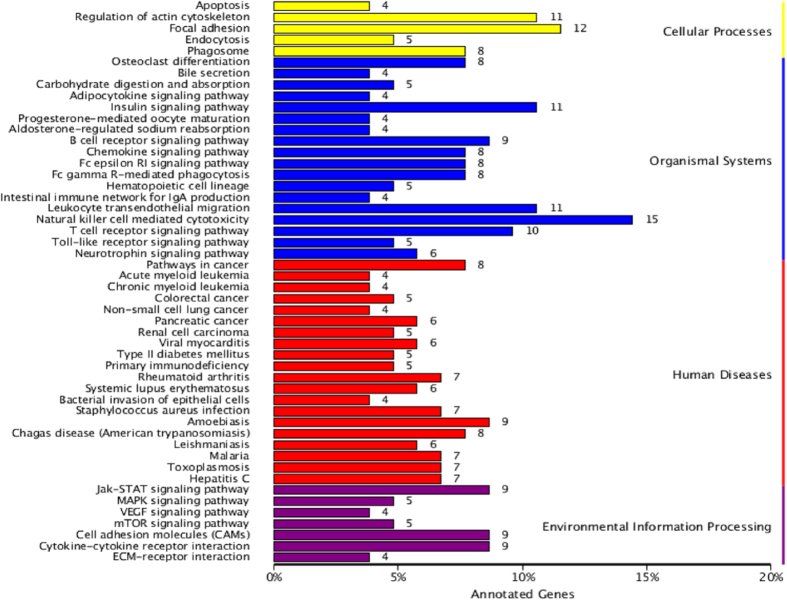
The enriched Kyoto Encyclopaedia of Genes and Genomes (KEGG) pathway of DEGs.

To gain further insight into the biological functions of the DEGs, GO analyses were preformed, including a biological process, a molecular function, and cell components. *P* < 0.05 was defined as the significance level for GO analyses. GO functional enrichment of DEGs and genome-wide assessments displayed the following four functional areas with large proportional differences: cell death phase, biological stage, cell aggregation stage, and receptor regulator activity. DAVID, an online program, was used for the up-regulation and down-regulation analysis of DEGs. The analysis results indicated that the functional pathway with the highest enrichment score involves the defense, inflammatory, and wound responses. Signal peptides, disulphide keys, glycoproteins, extracellular matrices, and glycosylation sites also had a relatively high enrichment (Fig. 9). A total of 140 DEGs and 50 metabolic pathways were enriched in the KEGG database (Fig. 10). In addition, the results indicated that a large number of genes were enriched in the signalling pathway related to stress, including the P53 signalling, TGFβ signalling, apoptosis signalling, and MAPK signalling pathways.

### Network analysis of DEGs

**Figure 11:**
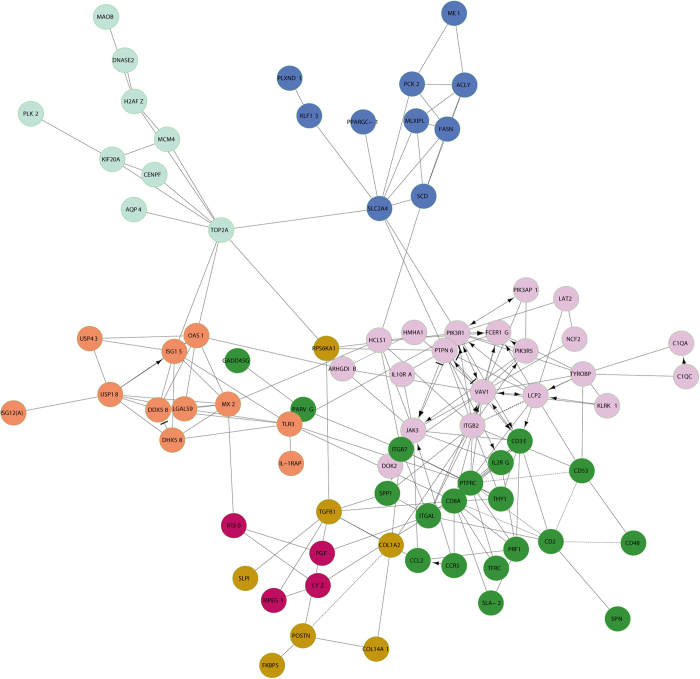
Functional interaction network of DEGs (FIs) in the cortisol group compared to the control group. The effect of interaction is represented by arrows, bar-headed lines, straight lines and imaginary lines. “→” for activating/catalysing, “-|” for inhibition, “-” for FIs that were extracted from complexes or inputs, and “---” for predicted FIs.

The Reactome plug-in in the Cytoscape software was used to visualise the functional interaction (FI) network of DEGs and to further identify major functional genes (Fig. 11). A number of DEGs, such as *TLR3*, *PTPRC*, *CCL2*, *TGFβ1*, *TOP2A*, *SPP1*, and *CD3ε* were key node genes. These genes may play important roles in the stress response. Furthermore, the *TLR*3, *CCL2*, *TGFβ1*, *SPP1, PTPRC*, *TOP2A* and *CD3ε* genes have been implicated in regulating calcium channels and cell apoptosis. There is a direct relationship between *TLR3* and *IL-1RAP*, which exhibit higher expression in the cortisol group compared to the control group that leads to nuclear factor kappa B (NF-κB) activation, which affects cell apoptosis.

## Discussion

Previous studies showed that stress activates the hypothalamus-pituitary-adrenal (HPA) axis and increases GC secretion and its receptors (GR), which are important factors of stress response. GR are involved in the regulation of food intake and energy balance. GR are up-regulated and played an essential role in the loss of appetite in the early stage of stress^22^,^23^. In the current study, the significantly increased serum cortisol concentration indicated that the pigs were in a stressed state, suggesting that cortisol may act on the GR through a series of genes and pathways in pigs and may lead to the deterioration of meat quality. There was no significant difference in weight between the two treatment groups, but compared to the control group, the cortisol group had slower weight gain. Body energy consumption is greatly increased when animals are subjected to high-intensity stress. The consumed energy relies on muscle glycogen fermentation to supplementation. When the supplementary energy is nearly exhausted, the activity of oxidative metabolism enzyme improves. Thus, stress causes a variety of metabolic changes independent of nutrient intake^24^. In addition, stress reduces the proportion of pigment to oxymyoglobin, resulting in loss of flesh brightness and dark colored flesh^25^. Our study demonstrates similar findings, such that with prolonged stress, the meat color lost brightness and became clearly darkened. On the other hand, drip loss is an indicator of muscle water properties. Leheska *et al*.^26^ reported that pigs that were transported 8 h had lower 24 h drip loss than pigs that were transported 0.5 h (*P* < 0.05)^26^. It was also reported that the drip loss at 24 h postmortem was decreased in a stress state. During the conversion of muscle to meat, lactic acid builds up in the tissue, leading to a reduction in the pH of the meat. After the pH reaches the isoelectric point of major proteins, the net charge of the protein is zero. The positive and negative charges within the protein are attracted to each other and result in a reduction in the amount of water^27^. In this experiment, compared to the control group, the drip loss of the cortisol group was significantly decreased (*P* < 0.01). Recently, it was reported that pre-slaughter bath and ventilation stress treatment in broiler chickens led to a significant decline in drip loss, which is consistent with our results^28^.

Proteins related to meat quality traits including CAST, MyoD1, Desmin, ATF4, IGF-1, calpain1, and calpain2, may play a crucial role in meat deterioration. An experiment on Hainan black goats investigated the effects of the nutritional levels of diets on meat quality, and the expressions levels of CAST and calpain1 were significantly affected^29^. Previous report provides a mechanistic link connecting calpain1, calpain2 with accelerated myofibrillar proteolysis^30^,^31^. In this study, high protein level of CAST, calpain1, and calpain2 resulted in lower drip loss, reducing meat tenderness after cortisol treatment. MyoD1, which belongs to the MRF family, initiates and maintains the differentiation and development of skeletal muscle^32^. MyoD1 plays important roles in meat quality, and over-expression of the MyoD1 inhibits the proliferation of myoblasts and promotes the differentiation of myoblasts to form mature muscle fiber cells^33^,^34^. In this experiment, compared to the control group, the protein expression of MyoD1 in the cortisol group was decreased and indicated that stress stimulation inhibits the expression of MyoD1, resulting in meat quality deterioration. Desmin is a key cytoskeletal protein, maintaining the structure of muscles^35^. A decrease in the level of Desmin is accompanied by an increase in drip loss and a decrease in shear force^36^. In this study, compared to the control group, a lower drip loss and lower protein expression of Desmin may correlate with an accelerated degradation of skeletal muscle. IGF-1 plays a major role in the regulation of skeletal muscle growth and improves the rate of protein synthesis and reduces the rate of protein degradation^37^. In this study, the protein expression level of IGF-1 in the cortisol group was lower than the control group, which indicated that the degradation rate of protein was enhanced, resulting in the deterioration of meat quality. It is reported that ATF4 is a skeletal muscle protein which can lead to the decline of meat quality during aging and a targeted reduction of ATF4 expression in skeletal muscle can represses muscle weakness^38^. Our results confirmed that increased protein expression of ATF4 caused a decrease in meat quality.

Serum cortisol concentration is an important index to determine whether pigs are in a stressed state. When animals are in a stressed state, external stimulation activates the HPA axis, resulting in an increase in GC secretion. Compared to the control group, the levels of cortisol in the serum were significantly increased (*P* < 0.01), which showed that the body was stressed in this study. However, blood glucose levels in the cortisol group were not affected. During transportation stress, blood glucose levels in pigs are significantly higher than the normal value, and the concentration significantly decreases with prolonged transportation time^39^. We therefore hypothesized that when pigs are under stress, the blood glucose level increases and then decreases, with blood glucose levels eventually being maintained a constant state. With the extension of stress time, stress physiological process regulated by pituitary-adrenocortical hormone lead to an increase in plasma GC and the enhancement of protein catabolism and gluconeogenesis, which results in constant blood glucose that resists stress.

When stimulation causes tissue damage, tissue enzymes penetrate the cell membrane and are released into the blood. Therefore, tissue enzymes can serve as an index for diagnosing stress. LDH can catalyze pyruvate so that glycolysis results, which produces lactic acid during glucose metabolism. The increase in LDH enzymatic activity is closely related to anaerobic glycolysis. There was no significant difference in the content of LDH in the blood between the cortisol group and control group, but the LDH content showed a decreasing trend. With the decrease in LDH activity, the energy metabolism activities of the organism are reduced. The LDH levels of the group subjected to heat stress treatment at different time were lower than the control group in Bama miniature pig, suggesting that heat stress reduces energy metabolism^40^. CK is an organ-specific enzyme, mainly existing in skeletal and cardiac muscle. The activity of CK in the blood may reflect the degree of damage to the related tissue cells. It was reported that the lairage duration and sex of pigs have an impact on meat quality at the CK level^41^. When animals were in a heat stress state, cell damaged and membrane permeability increased due to insufficient energy supply. Increased membrane permeability resulted in the release of CK, which in turn resulted in an increase in CK concentration in blood^42^. In contrast to Del Vesco *et al*.’s study, we found that the level of CK in the blood was significantly decreased in the stress state, although our finding is consistent with the results of another study showing that serum CK activity is increased (Day 1) and then reduced (Day 7–21) during heat stress of Bama miniature pigs^40^. A possible reason is that body repair stress resulted in a decline in the CK level with prolonged cortisol treatment time.

Stress led to a large gap between muscle fibers resulting in meat deterioration^43^. Our results revealed that the degree of damage and the muscle bundle ratio of the cortisol group was significantly higher than the control group. These results indicated that muscles are damaged most severely after stress. Another study reported that GC also mediates the apoptosis mechanism^44^. Our study revealed that more cell apoptosis and a higher apoptosis rate occurred in the cortisol group compared to the control group. Additionally, it was found that severe muscle injury and cell apoptosis in ducks postmortem have effects on muscle color and water holding capacity in stress state^45^. Apoptosis often occurs with damage to cytoskeleton proteins and cellular components and shrinkage of muscle cells, which leads to changes of meat quality traits^46^,^47^.

Zhang *et al*.^48^ reported that heat stress induced the mRNA and protein expression of BAX in LLC-PK1 cells, significantly increasing the ratio of Bcl-2 and BAX^48^. Other studies reported that knockout IP3R1 in T Jurkat lymphocytes prevented apoptosis^49^ and that apoptosis can also be prevented by blocking the SERCA1 protein^50^. Their studies indicated that higher levels of IP3R1 and SERCA1 may induce cell apoptosis. The imbalance of calcium homeostasis in the sarcoplasmic reticulum results in an increase of cytoplasmic calcium, leading to further activation of proteases and lipases that finally destroy the structure of cell membrane and cause apoptosis^51^. In our study, more cell apoptosis was found in the cortisol group than the control group, and the expression levels of calcium-channel-related genes (*SRECA1* and *IP3R1*) and apoptosis-related genes (*BAX*, *Bcl-2*, and *Caspase-3*) in the cortisol group were significantly higher than the control group (*P* < 0.05). Therefore, stress induces cell apoptosis by acting on calcium-channel-related genes. The death receptor pathway was reported to induce the cell apoptosis pathway through *Caspase-8* working on *Bid*, and then *Bid* acted on *Caspase-3* and *Caspase-7* to induce tumor cell apoptosis^52^.

We found that 223 DEGs were mainly enriched in the GO terms associated with the defense response, inflammation response, wound response, extracellular region, extracellular space, and extracellular region. Similar numbers of DEGs were identified from muscle tissue using sequencing techniques from previous studies^53^,^54^. In addition, previous studies showed that GC induces apoptosis and that calcium ion homeostasis has been implicated as a mediator of GC-induced apoptosis^55^. In this study, of these DEGs, *PTPRC*, *C5AR1*, *CCL2*, *STC1*, *TGFβ1* were related to calcium ion homeostasis, and *TLR3*, *PTPRC*, *CD3ε*, *SOX4*, *TGFβ1*, *TOP2A* were related to apoptosis regulation based on the DAVID database analysis. It is crucial to identify DEGs related to quality traits, which play an important role in muscle tissue^56^. We hypothesized that DEGs related to apoptosis and calcium ion homeostasis might have a major effect on reducing meat quality.

A large number of DEGs related to inflammation were found, such as *TLR3*, *IL-1RAP*, *TGFβ1*, *CCL2*, *SPP1*, and *GPX2*. These genes also influence cell apoptosis. Of 223 DEGs, *TLR3* and *IL-1RAP* of the cortisol group showed higher expression in skeletal muscle cells than those of control group. GC and TLRs were mutually regulated in a bidirectional manner. TLRs directly activated the release of adrenocortical steroid, indicating that TLRs might have an effect on the LD muscle as described in a previous study^57^. Furthermore, *TLR3* initiated the signal transduction of apoptosis to activate the caspase signal transduction pathway and ultimately to cause apoptosis^58^. The cytokine interleukin 1(IL-1) initiated a wide range of pro-inflammatory cascades, and its inhibition decreased inflammation in a variety of diseases^59^. Nuclear factor kappa B (NF-κB) is a transcription factor downstream of *TLR3* and *IL-1*. The “canonical” pathway for NF-κB activation is triggered by pro-inflammatory cytokines, such as *IL-1* and *TLR3*^60^,^61^. NF-κB activation occurred through the activation of cell surface receptors, such as *IL-1RAP* or *TLR3*, inducing cell apoptosis^62^. It could be concluded that GC induced apoptosis of skeletal muscle cells by affecting the *TLR3*, *IL-1RAP* and related signalling pathways.

**Figure 12:**
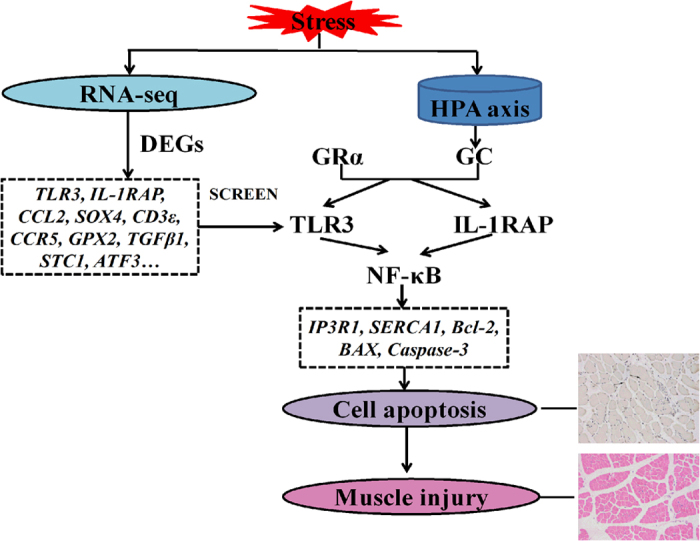
Molecular mechanism of effect of increased cortisol on meat quality deterioration based on transcriptome sequencing.

In summary, this study analyzed the meat quality index and blood biochemical index in a stressed state after cortisol was added to the pig diet, and it also determined the expression levels of genes related to calcium channels and cell apoptosis. RNA-Seq technology was used to analyze DEGs affecting meat quality in a stressed state and to screen key regulatory genes and pathways related to meat quality traits through enrichment analysis with the GO and KEGG database. These results indicated that *IP3R1*, *SERCA1*, *Bcl-2*, *BAX*, and *Caspase-3* were involved in the regulation of meat quality deterioration. Furthermore, CAST, MyoD1, ATF4, IGF-1, Desmin, calpain1, and calpain2 may be useful markers for meat quality trait in pigs after stress. A large number of DEGs related to calcium channels and cell apoptosis were screened, such as *TLR3*, *IL-1RAP*, *TGFβ1*, *CCL2*, *SPP1*, *GPX2*, *CD3ε*, *CCR5*, *ATF3*, and *STC1*. This study concluded that GC induces the apoptosis of skeletal muscle cells by affecting *TLR3* and *IL-1RAP*, which led to NF-κB activation. In addition, NF-κB also acted on *Caspase-3, Bcl-2*, and *BAX* to induce cell apoptosis, which led to changes in the normal physical and chemical properties of the muscle, ultimately resulting in deteriorated meat quality (Fig. 12). Our study focused on providing a theoretical basis for the internal mechanism of excessive stress during feeding and slaughter. Although a large number of DEGs were selected, the function of these genes and pathways and the effects of GC on meat quality decline remain to be studied.

## Materials and Methods

### Animals and samples

A total of 12 male piglets with the NN halothane genotype were used for the present study at 28 days old, and they had similar body weight. They were randomly assigned to the two treatment groups and raised in terms of the protocol issued by Huazhong Agricultural University. After 7 days of adaptation, the control group was fed a commercial diet (86% total digestible nutrients and 21.5% crude protein), and the cortisol group was fed the same diet with additional cortisol (hydrocortisone, Sigma-Aldrich, Inc., St. Louis, MO, USA) (120 mg/kg diet) for 7 days (Table 1). Piglets were fed with daily equalized food intake throughout the experimental period. Daily body weight and food intake were recorded during the 7 treatment days. At the age of 42 days, blood samples were collected from the jugular vein. LD were frozen immediately in liquid nitrogen and stored at −80 °C until used for RNA isolation. All of the studies involving animals were conducted according to the regulation (No. 5 proclamation of the Standing Committee of Hubei People’s Congress) approved by the Standing Committee of Hubei People’s Congress, P. R. China. The sample collection was approved by the Ethics Committee of Huazhong Agricultural University with the permit number No. 30700571 for this study. The animals were allowed access to feed and water ad libitum under the same normal conditions and were humanely sacrificed as necessary to ameliorate suffering. The methods were carried out in accordance with the approved guidelines.

### Determination of meat quality

Approximately 20 g of samples from the first and second lumbar LD were used to determine drip loss as a percentage of weight loss after 2 days of storage at 4 °C. Samples from the last lumbar LD were used for pH_45min_ and pH_24h_ determination across the sample surface with an electrode (pH-Star, Matthäus, Pöttmes, Germany). Samples from thoracolumbar LD were used for MC_45min_ and MC_24h_ determination with the Opto-Star (Matthäus)^63^. Infrared light is applied to the surface of meat from the Opto-Star, and the equipment obtains a color value by measuring the reflected light. Samples from the second to last ribs of the LD were used for EC_45min_ and EC_24h_ determination with the LF-Star (Matthäus).

### Determination of blood indexes

Blood was collected in 500-ml beakers and immediately separated into 10-ml tubes. Then, blood samples were centrifuged at 3,000× g for 10 min and stored at −80 °C for further use. Subsequently, the determination of cortisol was performed using immunosorbent assay kits purchased from ADL (American). The enzymatic activity of CK and LDH and the blood glucose concentration were determined using commercial kits purchased from Nanjing Jiancheng Biochemical Reagent Co., Nanjing (China) together with a biochemical analyzing machine (BT-224, Italy). Serum cortisol levels were measured using ELISA kits purchased from Elabscience (China) with an enzyme-labeled instrument (Multiskan MK3, American).

### Measurement of tissue morphology

Three samples were collected from the LD of each piglet, fixed in 10% formalin buffer, and stained with HE. The muscle bundle ratio was detected using HE staining. After the specific treatments, cells were grown on coverslips, and the apoptotic cell percentage of LD was detected using terminal deoxynucleotidyl transferase (TDT)-mediated dUTP digoxigenin nick-end labelling (TUNEL). Quantitative analysis of images was conducted using Image-Pro Plus6.0 software.

### Total RNA isolation and cDNA synthesis

Total RNAs were extracted from muscle by Trizol (Invitrogen, America) according to the manufacturer’s protocol. The RNA integrity was checked using denaturing gel electrophoresis and the RNA concentration was measured with a NanoDrop 2000 spectrophotometer (Thermo Scientific, Waltham). Total RNAs were reverse transcribed using a RevertAidTM First Strand cDNA Synthesis Kit (Fermentas, Lithuania).

### Quantitative Real-Time PCR

The expression levels of *GRα*, *IP3R1* and *SERCA1*, *Caspase-3*, *cytochrome C*, *Bcl-2*, and *BAX* were detected by quantitative real-time PCR (qRT-PCR). The qRT-PCR was performed with a Bio-Rad CFX384 Real-Time System using the SYBR Green PCR Master Mix (Bio-Rad, California) according to the manufacturer’s instructions. PCR detection was done in triplicate on 384 well plates. The expression level of *β-actin* was measured as an internal control, and *β-actin* was the reference gene for the qRT-PCR data^64^. All PCRs were performed in triplicate, and gene expression levels were quantified relative to the expression of *β-actin* with Gene Expression Macrosoftware (Bio-Rad) using a 2^−ΔΔCT^ value.

### mRNA sequencing and statistical analysis

Based on the synthesis of the side edge (sequencing by synthesis, SBS) technology, the HiSeq2500 Illumina high-throughput sequencing platform (Illumina, San Diego, CA, USA) was used to sequence a cDNA Library. A large number of high quality reads (raw data) was produced by removing the sequencing joints and primer sequence and by filtering low quality data^65^. After obtaining clean reads, TopHat2 was aligned with the reference genome (ftp://ftp.ncbi.nlm.nlh.gov/genomes/Sus_scrofa) to determine the positional information of the reference genome or gene and the characteristic information of the sequenced samples^66^.

### Functional annotation and enrichment analysis

Kyoto Encyclopaedia of Genes and Genomes (KEGG) pathway and Gene Ontology (GO) term analyses of these DEGs were performed using DAVID bioinformatics (http://david.abcc.ncifcrf.gov/) resources. Then, the mRNA regulatory network was constructed using the Cytoscape program. DESeq is applicable to experiment with biological repetition and can be used to analyze the differential expression between samples groups. During DEG detection, fold change ≥2, and *P* < 0.05 served as screening criteria.

### Identification of DEGs

The DEGs identified by the above-described method were validated using qRT-PCR. Primers were designed by using Primer-BLAST online primer design tool (http://www.ncbi.nlm.nih.gov/tools/primer-blast/). Samples were repeated 3 times. Relative genes expressions were calculated by using the 2^−ΔΔCT^ method and were analyzed.

### Immunohistochemistry

Sections were post-fixed for 20 min at room temperature with 4% paraformaldehyde (PFA; Sigma Aldrich, St. Louis, MO) that was freshly prepared and adjusted to pH 7.5. After three washes of 7 min each, the samples were permeabilized for 30 min using 0.2% Triton X-100(Sigma Aldrich, St. Louis, MO). Incubation with primary antibodies TLR3 (sc-12509, Santa Cruz Biotechnology) and IL-1RAP (ab8110, Abcam) was performed at room temperature for 2 h. DAB was used for visualization after incubation with the secondary antibody (IgG), and sections were then counterstained with haematoxylin and mounted for microscopic examination.

### Western Blot Analysis

Frozen muscles were homogenized in a Dounce homogenizer (Wheaton, Millville, NJ, 210) using RIPA lysis buffer (Beyotime, Nantong, China). Proteins (40 ug) were boiled in 5 × SDS buffer for 5 min, separated by SDS-PAGE (Bio-Rad, USA) on 12% gels and then transferred to polyvinylidene fluoride membranes (PVDF, Millipore, USA). After blocking with 5% fat-free milk in 0.1% TBS-T for 2 h, the membranes were incubated with primary antibodies against CAST (A7634, ABclonal), MyoD1 (A0671, ABclonal), Desmin (5332, CST), ATF4 (11815, CST), IGF-1(BA0939, Boster), calpain1 (ab28257, abcam), calpain2 (A1861, ABclonal), TLR3 (sc-12509, Santa Cruz Biotechnology), IL-1RAP (ab8110, Abcam), and GAPDH (ab8245, Abcam) overnight at 4 °C. After three 10-min washes in TBST, the membranes were incubated with the appropriate horseradish peroxidase-conjugated secondary antibodies for 2 h. After three 5-min washes in TBST, the bands were visualized with Super Signal West Pico Chemiluminescent Subs Kits (Pierce Biotechnology, Shanghai, China). Signals were quantitated using a Kodak Digital Sciences Image Station 440 (Eastman Kodak).

## Additional Information

**How to cite this article**: Wan, X. *et al*. Elucidating a molecular mechanism that the deterioration of porcine meat quality responds to increased cortisol based on transcriptome sequencing. *Sci. Rep*. **6**, 36589; doi: 10.1038/srep36589 (2016).

**Publisher’s note:** Springer Nature remains neutral with regard to jurisdictional claims in published maps and institutional affiliations.

## Supplementary Material

Supplementary Information

## Figures and Tables

**Table 1:** Different experimental diet administration.

Group	Treatment
Control (n = 6)	Commercial diet
Cortisol (n = 6)	Commercial diet + 120 mg/kg cortisol
