# Supplementary material for: Elucidating a molecular mechanism that the deterioration of porcine meat quality responds to increased cortisol based on transcriptome sequencing
Source: Sci Rep. 2016 Nov 11;6:36589. doi: 10.1038/srep36589 (PMC5105143; doi:10.1038/srep36589)
Supplement: Supplementary Information [file srep36589-s1.doc]

**Supplementary Information for:**

**Elucidating a molecular mechanism that the deterioration of porcine meat quality responds to increased cortisol based on transcriptome sequencing**

**Xuebin Wan ^1^, Dan Wang ^1^, Hong Xiang^1^, Huanan Li^1^, Hongshuai Wang^1^, Zezhang Liu^1^,Hongdan Niu ^1^, Jian Peng^2^, Siwen Jiang ^1^, Jin Chai ^1^,***

^1^Agricultural Ministry Key Laboratory of Swine Breeding and Genetics & Key Laboratory of Agricultural Animal Genetics, Breeding, and Reproduction of Ministry of Education, Huazhong Agricultural University, Wuhan, China

^2^Department of Animal Nutrition, Huazhong Agricultural University, Wuhan, China

*Correspondence to: J. Chai, College of Animal Science and Technology, Huazhong Agriculture University, Wuhan 430070, P.R. China. E-mail: [chaijin@mail.hzau.edu.cn](mailto:chaijin@mail.hzau.edu.cn);


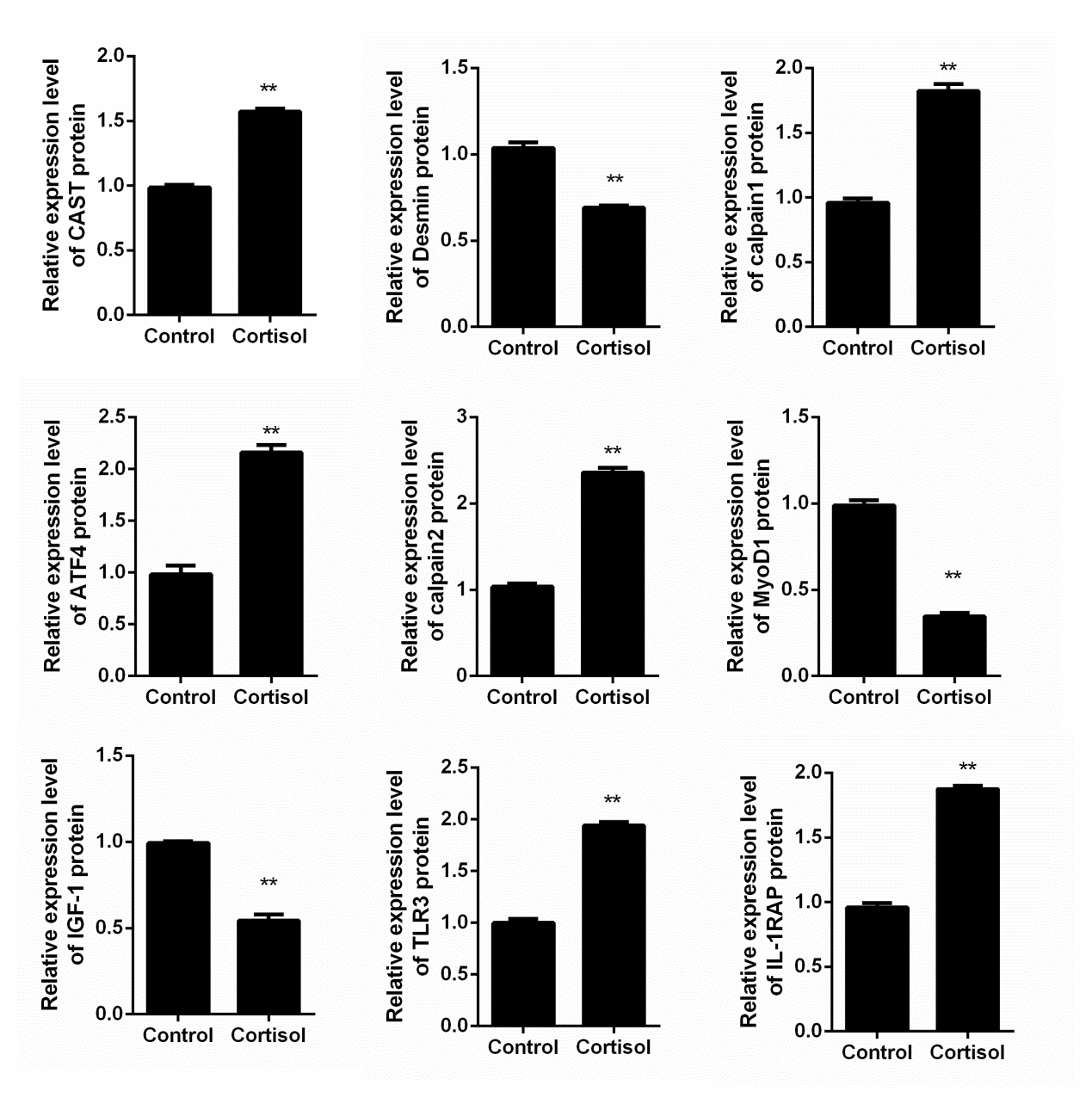


**Supplementary Figure S1: Quantification analysis of all of the western blot results.** Quantification

analysis of all of the western blot results demonstrated lower protein levels of Desmin, MyoD1, and IGF-1, and higher levels of CAST, calpain1, ATF4, calpain2, TLR3, and IL-1RAP in the cortisol group compared to the control group (**P* < 0.05, ** *P* < 0.01).


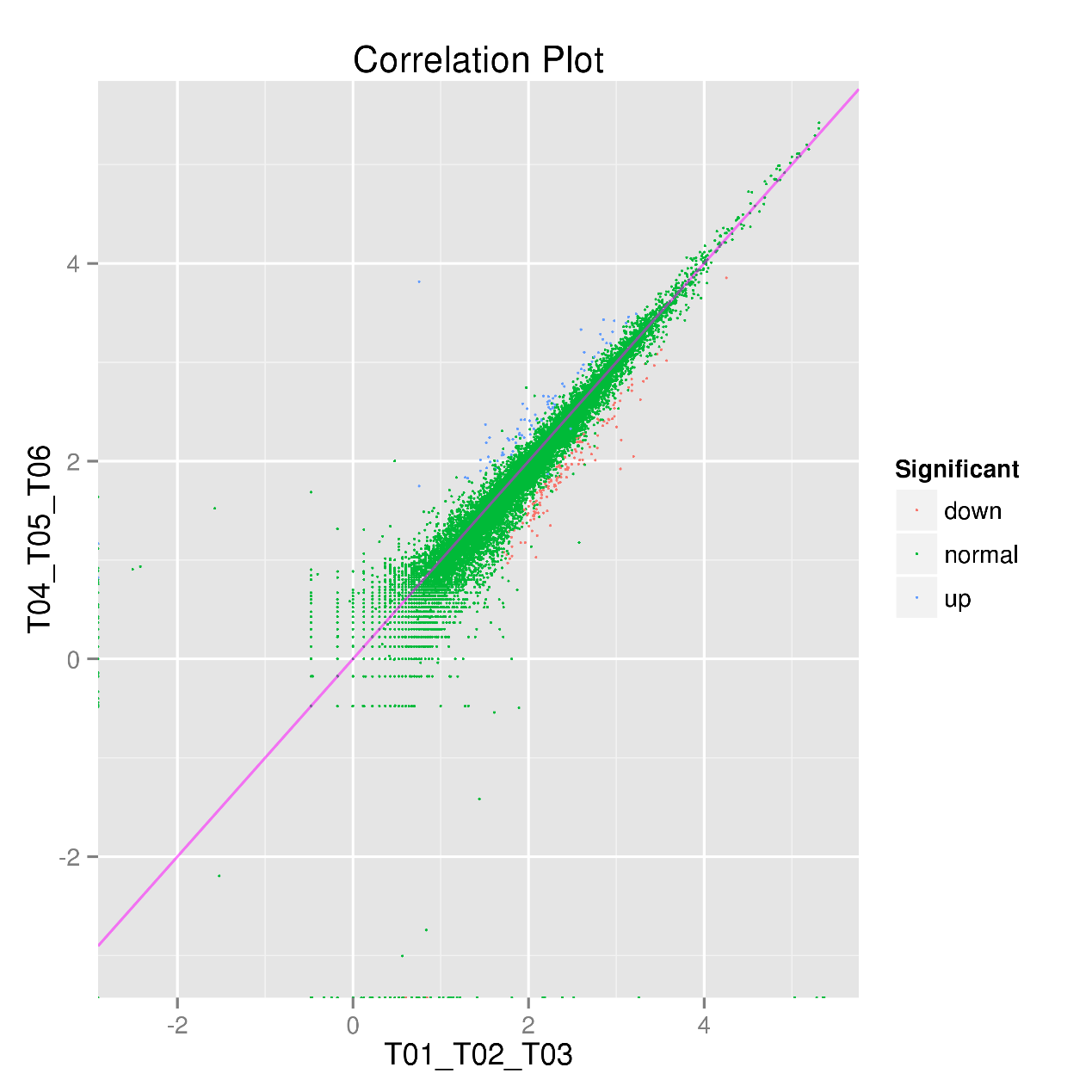


**Supplementary Figure S2: Scatter plots for the comparison of gene expression levels between two treatment groups.** Each point in the plot represents an individual gene. The more deviation from the diagonal, the higher expression level of the corresponding genes in the two samples. Control group (T01, T02, T03); Cortisol group (T04, T05, T06).

| **Supplementary Table S1. Alignment of clean data and reference genome** | | | | | |
| --- | --- | --- | --- | --- | --- |
| No. | Total Reads | Mapped Reads | Mapped Ratio | Uniq Mapped Reads | Uniq Mapped Ratio |
| T01 | 34,667,410 | 25,111,617 | 72.44% | 23,979,946 | 69.17% |
| T02 | 39,966,244 | 29,293,817 | 73.30% | 27,976,748 | 70.00% |
| T03 | 36,153,690 | 26,186,952 | 72.43% | 24,987,434 | 69.11% |
| T04 | 39,112,076 | 28,492,919 | 72.85% | 27,110,352 | 69.31% |
| T05 | 42,031,258 | 30,468,355 | 72.49% | 29,097,147 | 69.23% |
| T06 | 37,951,490 | 27,399,409 | 72.20% | 26,205,305 | 69.05% |

| **Supplementary Table S2. Differentially expressed genes (DEGs)** | | | | | | | | | | | |
| --- | --- | --- | --- | --- | --- | --- | --- | --- | --- | --- | --- |
| #ID | GeneName | Chr:strand:position | FDR | log2FC | regulate | T01 | T02 | T03 | T04 | T05 | T06 |
| ENSSSCG00000013517 | UHRF1 | (2):(-):(74678784-74711249) | 0.00232679 | -1.38987 | down | 3.303975105 | 4.187591737 | 1.94671317 | 1.4084259 | 1.327185512 | 0.866021203 |
| ENSSSCG00000026755 | KLF15 | (GL896227.2):(+):(46622-52812) | 0.00232679 | 1.60882 | up | 3.89522 | 2.197407512 | 2.889975 | 8.83843 | 9.09870514 | 9.46007 |
| ENSSSCG00000012974 | CFL1 | (2):(+):(5577568-5578984) | 0.00232679 | -1.07114 | down | 43.83754076 | 79.171361 | 46.938014 | 28.23147 | 24.274325 | 28.37924 |
| ENSSSCG00000027130 | TNFRSF12A | (3):(+):(40522157-40533352) | 0.00232679 | -1.51003 | down | 11.388459 | 57.47208 | 144.81171 | 27.44537 | 24.49073 | 23.08492 |
| ENSSSCG00000007978 | HBA | (3):(+):(40950681-40951551) | 0.00232679 | -1.04361 | down | 366.22786 | 156.630515 | 274.855021 | 267.198262 | 45.0453543 | 74.73774 |
| ENSSSCG00000030408 | DDX58 | (10):(+):(38145074-38192181) | 0.00605376 | -1.08459 | down | 2.221613 | 10.67089 | 1.398709 | 2.614234 | 2.21942 | 1.905039 |
| ENSSSCG00000016925 | PLK2 | (16):(-):(40067323-40073386) | 0.00232679 | -1.30631 | down | 6.652049 | 5.118195 | 6.112707 | 3.17958 | 2.089514 | 1.961938 |
| ENSSSCG00000015326 | COL1A2 | (9):(+):(81357192-81393120) | 0.00232679 | -1.19951 | down | 160.963 | 130.982 | 466.149 | 105.186 | 63.3884 | 161.516 |
| ENSSSCG00000030706 | FAM43B | (6):(+):(72981859-72982845) | 0.00950104 | 1.07058 | up | 4.84563 | 7.36969 | 2.735766 | 16.893386 | 6.35066 | 8.15739441 |
| ENSSSCG00000022913 | SLPI | (17):(+):(53167238-53169798) | 0.00232679 | 1.57756 | up | 25.15595 | 27.82595 | 47.6091 | 67.5923 | 134.7189 | 97.9165 |
| ENSSSCG00000027325 | TRNP1 | (6):(+):(77863824-77870220) | 0.00232679 | 1.55777 | up | 3.635852 | 1.884899 | 2.425998 | 14.56349 | 5.101852 | 3.72984 |
| ENSSSCG00000010850 | ENAH | (10):(-):(16218456-16292135) | 0.00950104 | -1.00437 | down | 8.516478207 | 13.74119598 | 38.58391509 | 16.68266 | 8.24277668 | 5.403374553 |
| ENSSSCG00000003017 | TGFB1 | (6):(-):(45127027-45139176) | 0.00232679 | -1.01329 | down | 10.91801 | 13.23136 | 14.75186 | 5.985945 | 6.125082 | 7.161205 |
| ENSSSCG00000011831 | APOD | (13):(+):(142076176-142094955) | 0.00232679 | 1.24078 | up | 12.555012 | 15.56222251 | 12.8183457 | 23.12772834 | 21.15097161 | 52.4626688 |
| ENSSSCG00000007816 | IL21R | (3):(-):(19681473-19722036) | 0.00425221 | -1.90653 | down | 2.724104506 | 1.691999 | 0.872224898 | 0.56379332 | 0.508301249 | 0.338476577 |
| ENSSSCG00000012832 | MXRA5 | (X):(-):(143912804-143940427) | 0.00232679 | -1.08526 | down | 7.666778 | 6.3120028 | 10.493954 | 3.713517474 | 2.754236 | 5.06642 |
| ENSSSCG00000029275 | PPARGC1 | (GL896199.1):(-):(15045-57267) | 0.00232679 | 1.34829 | up | 1.950473 | 1.956986 | 0.792196 | 4.579124676 | 4.953061 | 2.433606 |
| ENSSSCG00000024342 | AQP4 | (6):(-):(104383369-104394429) | 0.00232679 | 1.67927 | up | 8.6866 | 4.75934 | 5.49509 | 24.676 | 25.177 | 10.8087 |
| ENSSSCG00000021777 | NOVEL GENE | (2):(-):(50496893-50513253) | 0.00232679 | 1.16142 | up | 35.05999674 | 47.3640285 | 97.27673716 | 92.15354911 | 112.1143526 | 187.8733656 |
| ENSSSCG00000014084 | POC5 | (2):(-):(86324682-86366470) | 0.00232679 | 1.09259 | up | 2.186725 | 1.674731089 | 1.447132 | 2.053113883 | 4.929975 | 4.337825 |
| ENSSSCG00000016795 | BASP1 | (16):(+):(6829357-6866653) | 0.00232679 | -1.50728 | down | 3.36507205 | 10.27468664 | 5.23532 | 1.21466 | 1.684811686 | 3.740292894 |
| ENSSSCG00000010509 | PIK3AP1 | (14):(-):(117674451-117788256) | 0.00232679 | -1.7108 | down | 0.486168861 | 2.061758188 | 1.588856914 | 0.440989384 | 0.390592145 | 0.432164658 |
| ENSSSCG00000022307 | RAB7B | (9):(+):(72916107-72946419) | 0.00232679 | -2.39354 | down | 0.770296 | 1.97754 | 0.53091 | 0.178575 | 0.20693 | 0.23849 |
| ENSSSCG00000003524 | C1QA | (6):(+):(74609912-74612993) | 0.00232679 | -1.14177 | down | 21.73666 | 59.18563 | 63.8005 | 23.2934 | 17.671362 | 24.62411578 |
| ENSSSCG00000017983 | PER1 | (12):(-):(55836902-55851908) | 0.00232679 | 1.54742 | up | 16.7443759 | 14.631745 | 14.69553511 | 54.97955639 | 37.06629 | 42.619144 |
| ENSSSCG00000021742 | NOVEL GENE | (GL892316.1):(-):(25774-31862) | 0.00605376 | -1.54535 | down | 3.20546 | 4.82447 | 2.29551 | 1.67325 | 0.820283 | 1.04409 |
| ENSSSCG00000000138 | PVALB | (5):(+):(8553415-8573107) | 0.00232679 | -2.0082 | down | 350.83788 | 428.6934 | 216.7897 | 39.6343708 | 70.28721 | 137.74568 |
| ENSSSCG00000010464 | PPP1R3C | (14):(-):(112652687-112657270) | 0.00232679 | 1.09419 | up | 3.91528 | 6.22601 | 4.70695 | 18.5273 | 9.47639 | 0.757568 |
| Pig_newGene_6353 | NOVEL GENE | (GL896198.1):(+):(3257-7785) | 0.00232679 | -1.82177 | down | 35.6729 | 73.3691 | 27.87137 | 11.9816 | 19.62832 | 7.11921 |
| ENSSSCG00000010184 | AGT | (14):(-):(64380784-64389549) | 0.00425221 | 1.08173 | up | 7.14053 | 9.02952 | 9.45308 | 18.73935 | 18.21987 | 17.27409 |
| ENSSSCG00000003148 | NOVEL GENE | (6):(-):(49734283-49740069) | 0.00232679 | 1.26092 | up | 13.91325 | 5.95985 | 10.7544 | 26.97376 | 15.66914 | 30.75562 |
| ENSSSCG00000001770 | CTSH | (7):(-):(53623062-53644262) | 0.00232679 | -1.53324 | down | 16.868059 | 75.14219 | 47.02602 | 12.14577 | 14.21695 | 21.67448 |
| ENSSSCG00000029990 | PBD-2 | (15):(+):(43346318-43357071) | 0.00232679 | 10.2659 | up | 1.5518 | 1.36894 | 0.911356 | 3.49679 | 3.32171 | 4711.49 |
| ENSSSCG00000006357 | FCER1G | (4):(-):(97099150-97103132) | 0.00232679 | -1.41491 | down | 9.983707 | 58.18386 | 24.1581519 | 8.826335 | 9.52692 | 16.27182 |
| ENSSSCG00000007710 | CHREBP | (3):(-):(10598335-10616606) | 0.00232679 | 1.54455 | up | 0.815627614 | 0.934958361 | 0.608045327 | 2.399353 | 1.979077752 | 2.5020115 |
| ENSSSCG00000030730 | IGLV-4 | (14):(+):(52392752-52393255) | 0.00425221 |  | down | 1.03138 | 0 | 4.57867 | 0 | 0 | 0 |
| ENSSSCG00000008203 | IGKC | (3):(-):(59880799-59881322) | 0.00232679 | -2.30557 | down | 33.3529 | 106.5 | 144.795 | 8.14451 | 22.6819 | 32.7389 |
| ENSSSCG00000015581 | CENPF | (9):(-):(141697204-141740052) | 0.00232679 | -1.74798 | down | 0.718526 | 1.08625 | 0.579409 | 0.2955419 | 0.243734 | 0.17054 |
| ENSSSCG00000027196 | GIMAP6 | (18):(-):(6629467-6635745) | 0.00232679 | -1.64639 | down | 2.61450069 | 9.7818 | 7.4473 | 1.722562924 | 1.734544 | 2.881696019 |
| ENSSSCG00000010271 | PRF1 | (14):(-):(79362925-79368419) | 0.00425221 | -2.12099 | down | 0.871714 | 1.64929 | 1.60166 | 0.250141 | 0.400727 | 0.296887 |
| ENSSSCG00000016452 | GIMAP8 | (18):(-):(6894511-6906676) | 0.00232679 | -1.56367 | down | 0.542826413 | 3.485615587 | 2.355566 | 0.877957 | 0.555887 | 0.725799 |
| ENSSSCG00000010908 | PTPRC | (10):(+):(26308759-26332284) | 0.00232679 | -1.65304 | down | 3.06984997 | 8.29265 | 7.03459 | 2.06344808 | 0.9032983 | 2.882978 |
| ENSSSCG00000003644 | FHL3 | (6):(-):(86751333-86758637) | 0.00232679 | 1.46984 | up | 13.0242 | 13.3028 | 10.4335 | 28.7487 | 34.8772 | 38.197 |
| ENSSSCG00000000492 | LYZ | (5):(-):(36179190-36185575) | 0.00232679 | -2.62773 | down | 1.16650349 | 24.9482 | 3.57674 | 0.574565 | 1.384643 | 2.844835163 |
| ENSSSCG00000017421 | ACLY | (12):(+):(21082917-21106780) | 0.00232679 | 1.71598 | up | 2.865492 | 4.713315 | 3.510051 | 10.645628 | 4.364538 | 21.418778 |
| ENSSSCG00000025703 | NOVEL GENE | (1):(+):(86576990-86650762) | 0.00232679 | 1.35363 | up | 0.804153 | 1.05928 | 0.874029 | 2.07406 | 2.16028 | 2.76134 |
| ENSSSCG00000000774 | USP18 | (5):(+):(72536455-72596714) | 0.00232679 | -1.35054 | down | 4.54019 | 19.966 | 2.41964 | 3.00462 | 3.46381 | 4.09043 |
| ENSSSCG00000002432 | KCNK13 | (7):(+):(118431048-118546572) | 0.00425221 | 1.71947 | up | 0.312103607 | 0.51197013 | 0.987313 | 2.532841 | 0.669771 | 2.762568 |
| Pig_newGene_3867 | NOVEL GENE | (5):(-):(92762390-92771522) | 0.00232679 | 2.51708 | up | 0.402884692 | 1.017565 | 0.251865 | 2.690847201 | 4.44062 | 2.441216229 |
| ENSSSCG00000000257 | ITGB7 | (5):(-):(18830224-18839919) | 0.00232679 | -1.55381 | down | 2.103215 | 3.902129135 | 3.449248924 | 0.998215832 | 0.809981713 | 1.412128 |
| ENSSSCG00000026182 | NOVEL GENE | (1):(+):(296330290-296332085) | 0.00605376 | -1.0438 | down | 7.03835 | 27.3226 | 9.2156 | 8.65213 | 8.40706 | 4.07751 |
| ENSSSCG00000017473 | TOP2A | (12):(+):(22310214-22337485) | 0.00232679 | -1.47711 | down | 2.57301 | 3.32183 | 2.03654 | 1.36035 | 0.821117 | 0.66753 |
| ENSSSCG00000015122 | CD90 | (9):(-):(51638997-51644318) | 0.00232679 | -1.36996 | down | 64.0369 | 100.494039 | 102.909 | 29.5898 | 24.8344 | 49.0487 |
| ENSSSCG00000006800 | CD53 | (4):(-):(119674096-119703427) | 0.00232679 | -1.29226 | down | 3.129273886 | 13.4394 | 7.13325 | 3.202903918 | 1.91389 | 4.560977902 |
| ENSSSCG00000029715 | OLFM1 | (1):(+):(308316069-308353293) | 0.00232679 | 1.20084 | up | 1.641477 | 10.04725 | 3.43784 | 16.69735 | 11.9818 | 6.0927 |
| ENSSSCG00000000641 | KLRK1 | (5):(+):(64370014-64378469) | 0.00425221 | -1.6835 | down | 2.847663 | 10.54531 | 6.2157 | 2.277206287 | 0.928412083 | 2.899093 |
| ENSSSCG00000002009 | PCK2 | (7):(-):(80458471-80468943) | 0.00232679 | -1.5718 | down | 7.12941 | 3.07493 | 1.11985 | 1.88902 | 1.51278 | 0.407534 |
| ENSSSCG00000005494 | NOVEL GENE | (1):(-):(286732772-286813962) | 0.00232679 | -1.83018 | down | 2.388997 | 12.204781 | 5.80300836 | 0.929453177 | 1.682645076 | 3.124095886 |
| ENSSSCG00000005636 | SLC25A25 | (1):(+):(302453222-302567491) | 0.00232679 | 1.04485 | up | 35.33573 | 44.81672 | 32.35758 | 106.67912 | 52.29535899 | 73.15044 |
| ENSSSCG00000021943 | NOVEL GENE | (GL896296.2):(-):(4933-5477) | 0.00232679 | -1.16105 | down | 626.456 | 477.021 | 1811.92 | 385.141 | 248.381 | 670.205 |
| ENSSSCG00000029691 | CORO1A | (GL892962.1):(+):(1451-5468) | 0.00232679 | -1.77861 | down | 4.85714 | 15.7271 | 9.33692 | 2.26909 | 2.15972 | 4.29215 |
| ENSSSCG00000014395 | PCDH12 | (2):(-):(149752166-149765761) | 0.00232679 | -1.21052 | down | 8.53796 | 3.981911 | 6.228648081 | 2.90277742 | 2.972299421 | 2.2263841 |
| ENSSSCG00000027565 | HMHA1 | (2):(+):(77642049-77655498) | 0.00232679 | -1.17154 | down | 3.00294 | 7.56726 | 5.24372 | 1.492679399 | 2.45632463 | 3.07152 |
| ENSSSCG00000016958 | PIK3R1 | (16):(-):(50367103-50379440) | 0.00232679 | 1.42739 | up | 5.486086131 | 10.61139214 | 7.037773 | 24.862497 | 15.98574702 | 21.3764435 |
| ENSSSCG00000009612 | DOK2 | (14):(-):(6618168-6622585) | 0.00232679 | -1.67164 | down | 1.378304624 | 8.88024 | 3.921261 | 1.0102681 | 1.014252 | 2.426477 |
| ENSSSCG00000024899 | FAM101B | (12):(-):(49514985-49525727) | 0.00232679 | -1.1202 | down | 12.7046292 | 3.124124305 | 8.82158 | 6.164396329 | 2.8259219 | 2.349577 |
| ENSSSCG00000010123 | CLDN5 | (14):(-):(54778678-54779334) | 0.00232679 | -1.17551 | down | 46.9651 | 20.3778 | 30.6083 | 18.3429 | 9.23519 | 15.7876 |
| ENSSSCG00000017991 | PIK3R5 | (12):(-):(56731612-56799399) | 0.00950104 | -1.26374 | down | 1.097352127 | 1.966765732 | 1.537557161 | 0.572582798 | 0.630499843 | 0.713340056 |
| ENSSSCG00000008245 | TMSB10 | (3):(-):(62820737-62821926) | 0.00232679 | -1.11269 | down | 401.58 | 503.089 | 449.244 | 179.427 | 183.444 | 263.22 |
| ENSSSCG00000009882 | OAS1 | (14):(+):(41260566-41268627) | 0.00784602 | -1.40877 | down | 26.6476 | 75.087 | 12.5454 | 15.5304 | 15.586 | 11.9251 |
| ENSSSCG00000015085 | IL10RA | (9):(+):(50364900-50379299) | 0.00232679 | -1.29476 | down | 0.962787611 | 3.706196067 | 6.399491 | 1.332191906 | 0.99700114 | 2.182350823 |
| ENSSSCG00000027357 | CSTB | (GL896515.1):(-):(734-1607) | 0.00232679 | -1.237 | down | 37.9383 | 164.33 | 47.2032 | 27.0237 | 37.4996 | 41.3154 |
| ENSSSCG00000027487 | LAT2 | (3):(+):(11037022-11055319) | 0.00232679 | -1.33911 | down | 3.024506 | 16.3139 | 6.65341 | 2.58519675 | 2.942577 | 4.745868 |
| ENSSSCG00000017006 | LCP2 | (16):(+):(58039300-58082197) | 0.00232679 | -1.44709 | down | 1.290923134 | 4.860338773 | 3.63264 | 1.150968448 | 0.993662 | 1.4437072 |
| ENSSSCG00000009412 | LCP1 | (11):(+):(21569933-21685279) | 0.00232679 | -1.87501 | down | 4.561661 | 25.15679 | 10.10916 | 3.5841039 | 2.57206565 | 4.70186 |
| ENSSSCG00000021206 | IL1RAP | (13):(+):(137315798-137369721) | 0.00425221 | 1.77111 | up | 0.265364003 | 0.401562919 | 0.334292254 | 0.930252 | 1.205439 | 1.281652018 |
| ENSSSCG00000030543 | COTL1 | (GL895339.1):(-):(14934-61203) | 0.00232679 | -2.33682 | down | 8.49523 | 36.768 | 10.1209 | 2.15072 | 3.65478 | 5.15759 |
| ENSSSCG00000025537 | PTPN6 | (5):(-):(66198251-66208731) | 0.00232679 | -1.46007 | down | 2.29627 | 11.8643 | 6.49599 | 1.9228 | 2.42392 | 3.16145 |
| ENSSSCG00000001472 | SLA | (7):(-):(29545479-29549278) | 0.00425221 | -1.24319 | down | 3.93329 | 3.37756 | 5.08455 | 1.75107 | 2.25312 | 1.23208 |
| ENSSSCG00000003593 | NKAIN1 | (6):(-):(81479841-81487848) | 0.00232679 | 1.81142 | up | 2.456293412 | 4.779852395 | 3.256872634 | 12.58697366 | 14.11564985 | 10.12648348 |
| ENSSSCG00000026753 | OSBPL11 | (13):(+):(144365411-144416364) | 0.00784602 | 1.07841 | up | 2.64205 | 2.49423 | 2.27469 | 4.97433 | 5.16345 | 5.512 |
| ENSSSCG00000015801 | TLR3 | (15):(-):(53840783-53852536) | 0.00232679 | 5.40254 | up | 1.100563 | 1.394952 | 0.777690435 | 0.838704 | 114.0306241 | 23.58264065 |
| ENSSSCG00000028322 | BTG2 | (9):(+):(70327445-70331400) | 0.00232679 | -1.51849 | down | 41.7343 | 20.2811 | 109.582 | 6.57784 | 45.6599 | 7.65849 |
| ENSSSCG00000008239 | CAPG | (3):(-):(62357528-62367116) | 0.00232679 | -1.71019 | down | 7.32618331 | 43.6185773 | 15.84689277 | 4.003126173 | 8.39367949 | 8.01610639 |
| ENSSSCG00000008722 | SH3TC1 | (8):(-):(4324662-4354206) | 0.00784602 | -1.47722 | down | 0.971299195 | 0.565242147 | 1.234795053 | 0.375879511 | 0.267013294 | 0.35251579 |
| ENSSSCG00000024344 | CCR5 | (13):(+):(32547168-32551732) | 0.00232679 | -1.67026 | down | 0.949991 | 5.31327 | 4.71803 | 1.14916 | 0.302757 | 1.99836 |
| ENSSSCG00000017933 | SLC2A4 | (3):(+):(139903946-139910176) | 0.00232679 | 1.04208 | up | 23.02804 | 11.01252 | 14.82041 | 34.90234 | 30.42177 | 35.29043 |
| Pig_newGene_3489 | NOVEL GENE | (4):(+):(89843028-89847752) | 0.00232679 | 3.60736 | up | 0 | 0 | 0 | 0.392225 | 1.10758 | 0.137555 |
| ENSSSCG00000006273 | MCM4 | (4):(-):(87134012-87185073) | 0.00232679 | -1.18716 | down | 3.2515431 | 5.75961 | 2.19314009 | 1.887877749 | 1.30720109 | 1.725444271 |
| ENSSSCG00000008647 | CMPK2 | (3):(+):(137688568-137704282) | 0.00232679 | -1.02711 | down | 6.656213 | 15.93284 | 3.044971385 | 4.9211191 | 4.64139473 | 3.0159028 |
| ENSSSCG00000015617 | G0S2 | (9):(+):(146493481-146494458) | 0.00232679 | 1.56952 | up | 3.662526 | 11.45268 | 5.477903 | 37.24524 | 18.60125 | 5.274909 |
| ENSSSCG00000003231 | NKG7 | (6):(-):(51692516-51695384) | 0.00232679 | -1.62124 | down | 5.54552 | 23.088753 | 11.049655 | 4.08291 | 2.69348 | 6.12307 |
| ENSSSCG00000022236 | FOLR1 | (9):(+):(7507499-7511916) | 0.00232679 | -1.30052 | down | 17.1045 | 68.2691 | 53.9678 | 15.4161 | 15.9313 | 25.2224 |
| ENSSSCG00000010578 | PITX3 | (14):(-):(123084897-123086505) | 0.00425221 | 1.17484 | up | 7.68551 | 4.29477 | 5.10211 | 15.94231 | 11.27447829 | 11.34986 |
| ENSSSCG00000017927 | BCL6B | (12):(-):(54708169-54711360) | 0.00232679 | -1.01207 | down | 11.03022418 | 3.328461 | 8.140331 | 4.231217416 | 3.94079333 | 2.9837746 |
| ENSSSCG00000010806 | RGS2 | (10):(-):(2543423-2546894) | 0.00232679 | -1.28178 | down | 5.34913 | 12.9629 | 7.80775 | 3.47441 | 2.24185 | 5.0265 |
| ENSSSCG00000021006 | NOVEL GENE | (GL896422.1):(+):(1698-3396) | 0.00232679 | -3.32045 | down | 6.40691 | 11.39685 | 11.2329 | 0.632536 | 1.486191123 | 0.787909549 |
| ENSSSCG00000009178 | H2AFZ | (8):(+):(129743691-129745568) | 0.00425221 | -1.05022 | down | 24.4589 | 35.9782 | 19.321 | 15.143 | 13.269 | 10.1028 |
| ENSSSCG00000025034 | LAPTM5 | (6):(-):(81070177-81095159) | 0.00232679 | -1.42432 | down | 13.1582 | 45.6531 | 32.4512 | 11.28972 | 6.587645 | 16.1266 |
| ENSSSCG00000002376 | PGF | (7):(-):(103994328-104006524) | 0.00232679 | 1.32628 | up | 2.92827378 | 3.536234 | 2.276194 | 7.308987556 | 6.6779098 | 7.93088 |
| ENSSSCG00000008939 | IGJ | (8):(-):(71347284-71358511) | 0.00605376 | -2.17877 | down | 6.615 | 11.46636346 | 12.73911997 | 2.285626677 | 2.805972561 | 1.715534837 |
| ENSSSCG00000006717 | PHGDH | (4):(-):(111436317-111468486) | 0.00232679 | -1.63645 | down | 26.16684 | 4.677411 | 4.09021 | 4.183153 | 4.190889 | 2.86251 |
| ENSSSCG00000012397 | IL2RG | (X):(-):(64673721-64677978) | 0.00232679 | -1.83925 | down | 2.53728843 | 11.41111741 | 4.491405662 | 1.444316042 | 1.413308721 | 2.295689063 |
| ENSSSCG00000003525 | C1QC | (6):(+):(74616233-74621248) | 0.00232679 | -1.26742 | down | 15.47955 | 44.739126 | 53.202887 | 23.808251 | 17.20474 | 25.2909 |
| ENSSSCG00000011133 | PFKFB3 | (10):(+):(70831452-70853803) | 0.00232679 | -2.09105 | down | 35.131558 | 10.013354 | 7.660597036 | 3.363200777 | 6.108394 | 2.92239 |
| Pig_newGene_1230 | NOVEL GENE | (13):(+):(111049460-111050548) | 0.00232679 | -1.28129 | down | 18.2802 | 64.5579 | 14.3541 | 12.7037 | 16.6552 | 10.6287 |
| ENSSSCG00000001549 | FKBP5 | (7):(-):(36373069-36478480) | 0.00232679 | 2.72929 | up | 6.330314 | 9.297699431 | 3.8030201 | 47.55646 | 34.898523 | 46.397717 |
| ENSSSCG00000002389 | VASH1 | (7):(+):(105960537-105978240) | 0.00232679 | -1.58561 | down | 9.91505 | 3.80272 | 6.11475 | 3.13488 | 1.44729 | 2.02568 |
| ENSSSCG00000013513 | PLIN5 | (2):(+):(74797981-74806557) | 0.00232679 | 1.26401 | up | 11.409456 | 3.845369793 | 4.0784432 | 22.30217 | 17.75919 | 6.36976229 |
| ENSSSCG00000001703 | NFKBIE | (7):(-):(45124044-45132794) | 0.00605376 | -1.33002 | down | 3.210603 | 8.39928 | 6.07274 | 1.278499 | 2.92229 | 2.832696 |
| ENSSSCG00000007366 | MYBL2 | (17):(+):(51696566-51726535) | 0.00232679 | -1.47458 | down | 4.3275474 | 6.092907 | 2.8026467 | 2.03561 | 1.3564 | 1.36617 |
| ENSSSCG00000009240 | PLAC8 | (8):(+):(144545691-144573889) | 0.00425221 | -1.25185 | down | 4.01306 | 29.892136 | 5.7327 | 4.40229 | 3.50505 | 8.73696 |
| ENSSSCG00000008535 | CLIP4 | (3):(-):(116933719-117001691) | 0.00232679 | 1.03195 | up | 1.88934515 | 2.101852126 | 1.38020909 | 4.34180644 | 2.76438351 | 3.877198292 |
| ENSSSCG00000006140 | CA2 | (4):(-):(56156732-56174841) | 0.00232679 | 1.44375 | up | 1.0198 | 2.07203 | 2.31495 | 5.06146 | 5.29427 | 4.35215 |
| ENSSSCG00000015270 | FMOD | (9):(-):(70356564-70367608) | 0.00232679 | -1.50003 | down | 14.6436 | 7.85367 | 30.872 | 4.59667 | 4.04762 | 10.2242 |
| ENSSSCG00000009642 | STC1 | (14):(-):(8805561-8816800) | 0.00232679 | 1.48999 | up | 1.185854 | 0.895192 | 0.702315 | 3.6442 | 1.414802 | 2.7591 |
| Pig_newGene_1744 | NOVEL GENE | (14):(-):(47943782-47951144) | 0.00232679 | 1.23811 | up | 14.065 | 10.4629 | 5.77426 | 27.2247 | 24.9612 | 15.4564 |
| Pig_newGene_5878 | NOVEL GENE | (GL894379.1):(-):(1-460) | 0.00232679 | -1.68297 | down | 1202.77 | 1762.55 | 1337.14 | 368.06 | 594.595 | 377.301 |
| ENSSSCG00000003558 | RPS6KA1 | (6):(+):(77442985-77479999) | 0.00784602 | -1.18441 | down | 1.634019623 | 5.84955 | 4.128697 | 1.780391568 | 1.668103814 | 1.660949265 |
| ENSSSCG00000026302 | MKI67 | (14):(-):(149170562-149198585) | 0.00232679 | -1.77554 | down | 2.063115 | 3.98975715 | 1.479306414 | 0.867237922 | 0.609606897 | 0.723183402 |
| ENSSSCG00000017391 | PLEKHH3 | (12):(-):(20365602-20374639) | 0.00425221 | 1.11467 | up | 9.846636 | 10.227585 | 7.536362 | 32.12218 | 14.050987 | 13.616459 |
| ENSSSCG00000020953 | ATP1A4 | (4):(-):(98232795-98273614) | 0.00232679 | 3.01455 | up | 0.903268 | 0.78676 | 0.598685 | 4.8694 | 7.54952 | 6.07635 |
| ENSSSCG00000002648 | CBFA2T3 | (6):(+):(1022607-1044687) | 0.00232679 | 1.28328 | up | 6.168595 | 8.615957 | 5.95822915 | 23.43358 | 12.686765 | 14.365994 |
| ENSSSCG00000013731 | DNASE2 | (2):(-):(66447489-66450524) | 0.00605376 | -1.31112 | down | 1.70448139 | 8.41187 | 3.97995 | 1.74149509 | 1.51159 | 2.42784 |
| ENSSSCG00000000654 | CLECL1 | (5):(+):(64938961-64956629) | 0.00232679 | -1.87809 | down | 1.512892079 | 17.91364 | 3.729355 | 1.902886 | 1.583172 | 2.81337 |
| ENSSSCG00000029944 | FASN | (12):(-):(1023477-1034681) | 0.00232679 | 1.67916 | up | 6.16205 | 7.421712 | 18.6239 | 33.432706 | 11.2609 | 58.4484 |
| ENSSSCG00000017723 | CCL2 | (12):(+):(42514168-42516128) | 0.00425221 | -1.98758 | down | 2.90963 | 13.5086 | 4.1785 | 1.2704 | 1.9567 | 1.96659 |
| ENSSSCG00000007797 | ITGAL | (3):(+):(18259532-18298947) | 0.00950104 | -1.03559 | down | 1.884319162 | 4.11694615 | 3.2576085 | 1.500876 | 1.341083023 | 1.674674 |
| ENSSSCG00000013901 | IFI30 | (2):(+):(59214055-59218018) | 0.00232679 | -1.45648 | down | 12.2121 | 49.8955 | 27.6209 | 6.70518 | 12.1312 | 13.8589 |
| ENSSSCG00000023044 | NOVEL GENE | (12):(-):(1048559-1052166) | 0.00232679 | 1.07679 | up | 21.22972 | 41.0204 | 65.2664 | 71.94914 | 42.5789 | 154.4488 |
| ENSSSCG00000006979 | MSR1 | (17):(-):(4110396-4191497) | 0.00232679 | -2.24258 | down | 0.536110844 | 9.80293 | 1.792322151 | 1.00127567 | 0.78582 | 0.776357 |
| ENSSSCG00000009361 | POSTN | (11):(-):(13198182-13233797) | 0.00232679 | -1.33741 | down | 6.54235 | 7.195259 | 25.59343 | 4.90653 | 2.225755 | 8.432202 |
| ENSSSCG00000002831 | IRX3 | (6):(+):(28168074-28170875) | 0.00232679 | 1.29902 | up | 2.17898 | 2.9165 | 4.60954 | 11.2536 | 7.693 | 4.9338 |
| ENSSSCG00000016451 | GIMAP1 | (18):(+):(6872941-6875292) | 0.00232679 | -1.49219 | down | 6.37075 | 29.7551 | 16.313 | 4.49408 | 5.10584 | 9.04071 |
| ENSSSCG00000027826 | GIMAP4 | (18):(+):(6618395-6619630) | 0.00232679 | -1.61108 | down | 3.005556 | 26.016 | 9.852997 | 4.42563 | 3.5194 | 4.780633165 |
| ENSSSCG00000017754 | LGALS9 | (12):(+):(46002258-46021360) | 0.00232679 | -1.43719 | down | 23.77646 | 69.9961 | 14.51697 | 14.09439 | 12.55465 | 13.34065 |
| ENSSSCG00000011592 | PLXND1 | (13):(-):(76179149-76229571) | 0.00232679 | -1.22195 | down | 22.250026 | 10.42459023 | 17.17805 | 8.77795 | 5.749327 | 6.844786546 |
| ENSSSCG00000009955 | CRYBB2 | (14):(+):(46033975-46043586) | 0.00232679 |  | up | 0 | 0 | 0 | 8.0262 | 0 | 0 |
| ENSSSCG00000011884 | HCLS1 | (13):(+):(148480008-148508215) | 0.00232679 | -1.29239 | down | 5.46351 | 13.039866 | 8.58582 | 3.322903 | 3.75162 | 3.9853 |
| ENSSSCG00000015436 | CCDC71L | (9):(-):(117342025-117342987) | 0.00232679 | 1.65641 | up | 2.26684 | 2.88478 | 1.690958 | 7.93217 | 5.983212 | 7.65449 |
| ENSSSCG00000021944 | RAC2 | (5):(+):(8149172-8167615) | 0.00232679 | -1.55023 | down | 2.62254 | 8.04384 | 5.532564 | 1.67226 | 1.527793366 | 2.33116 |
| ENSSSCG00000027982 | ISG15 | (GL895967.2):(+):(58449-59139) | 0.00232679 | -1.85572 | down | 3.07995 | 4.21853 | 1.04622 | 0 | 2.22547 | 3.03711 |
| ENSSSCG00000027348 | WIPF1 | (15):(-):(89761503-89836721) | 0.00605376 | -1.0102 | down | 4.91203 | 7.472755979 | 6.973965629 | 3.649680903 | 3.05695 | 2.904584119 |
| ENSSSCG00000006579 | S100A3 | (4):(-):(104909992-104915210) | 0.00425221 | -2.47699 | down | 1.31224092 | 8.41086287 | 3.74763131 | 1.08310813 | 0.94828211 | 1.48988284 |
| ENSSSCG00000003647 | NOVEL GENE | (6):(+):(86858438-86865608) | 0.00232679 | 1.4448 | up | 9.50246 | 10.7155 | 6.90737 | 20.8675 | 26.3103 | 26.6642 |
| ENSSSCG00000003720 | NOVEL GENE | (6):(+):(104601216-104604724) | 0.00232679 | 2.0312 | up | 20.7605 | 13.21052 | 16.14975 | 68.6764 | 90.3498 | 45.8396 |
| ENSSSCG00000017995 | USP43 | (12):(+):(57342299-57399793) | 0.00232679 | 1.42374 | up | 0.314143971 | 0.432369015 | 0.558268963 | 0.833396 | 0.88823179 | 1.77883404 |
| ENSSSCG00000026931 | SERTAD1 | (6):(-):(44447547-44448266) | 0.00232679 | 1.16606 | up | 4.711786 | 9.09703 | 9.58097 | 32.71177 | 17.53576 | 19.60945 |
| ENSSSCG00000029371 | C5AR1 | (6):(+):(48523114-48537524) | 0.00232679 | -1.40126 | down | 0.684504 | 5.35157 | 5.8102 | 1.08054 | 1.71954 | 1.68492 |
| ENSSSCG00000011848 | TFRC | (13):(+):(143408460-143437991) | 0.00232679 | 1.3464 | up | 11.30843769 | 14.77634138 | 10.393504 | 30.46204956 | 38.35589072 | 23.937499 |
| ENSSSCG00000000604 | ARHGDIB | (5):(+):(60965351-60987579) | 0.00232679 | -1.5733 | down | 17.3889 | 49.1446 | 24.58363 | 9.96184 | 9.11306 | 11.54409 |
| ENSSSCG00000014048 | HK3 | (2):(+):(82651078-82669061) | 0.00425221 | -1.8004 | down | 1.907704283 | 11.688853 | 1.613469822 | 1.16084468 | 1.6446898 | 1.56117894 |
| ENSSSCG00000025136 | NOVEL GENE | (2):(+):(4826321-4839557) | 0.00232679 | 2.29897 | up | 2.650639756 | 1.605436742 | 2.843923166 | 13.19125132 | 18.99294804 | 2.755181688 |
| ENSSSCG00000012652 | SASH3 | (X):(+):(122142844-122158078) | 0.00232679 | -2.09097 | down | 1.939027 | 6.70929 | 4.40716 | 0.919902 | 0.732408275 | 1.412101 |
| ENSSSCG00000026006 | KLF13 | (1):(-):(159396562-159485685) | 0.00232679 | 1.13703 | up | 8.96392 | 6.02106 | 7.22037 | 19.9467 | 12.182 | 16.707 |
| ENSSSCG00000030820 | STK4 | (17):(-):(52943152-52982857) | 0.00232679 | -1.25017 | down | 1.07019 | 3.02331 | 2.55297 | 1.11627 | 0.90077 | 0.777128 |
| ENSSSCG00000016501 | KDM7A | (18):(+):(10087542-10164670) | 0.00232679 | -1.34279 | down | 6.63825 | 4.13969 | 6.63881 | 2.24413 | 2.64643 | 1.97614 |
| ENSSSCG00000005997 | COL14A1 | (4):(-):(19715674-19949617) | 0.00232679 | -1.04685 | down | 10.75137109 | 11.72533 | 47.501493 | 11.445981 | 6.0068817 | 16.418225 |
| ENSSSCG00000026478 | PADI2 | (6):(-):(70039586-70099223) | 0.00232679 | -1.79582 | down | 0.84742 | 2.24798 | 1.34247 | 0.290425 | 0.393696 | 0.594018 |
| ENSSSCG00000009004 | SFRP2 | (8):(+):(79743275-79751980) | 0.00605376 | -1.06121 | down | 7.8801 | 110.708 | 27.809 | 16.3663 | 7.18554 | 46.6061 |
| ENSSSCG00000013116 | TCN1 | (2):(-):(11135823-11136343) | 0.00425221 |  | down | 0 | 6.60777 | 0 | 0 | 0 | 0 |
| ENSSSCG00000030095 | ZBTB16 | (9):(+):(46458177-46459448) | 0.00232679 | 1.34489 | up | 0.509136 | 1.24595 | 1.31344 | 11.1789 | 0.00507906 | 9.90118 |
| ENSSSCG00000004454 | ME1 | (1):(+):(93076563-93242655) | 0.00232679 | 1.16523 | up | 2.100399469 | 3.483563994 | 2.680826576 | 4.15394251 | 5.049804 | 9.33162236 |
| ENSSSCG00000013553 | VAV1 | (2):(-):(72827624-72857071) | 0.00232679 | -1.66539 | down | 1.01122 | 5.38832 | 3.25394 | 0.904967 | 0.835491 | 1.30288 |
| ENSSSCG00000016892 | FST | (16):(+):(34778435-34785305) | 0.00232679 | 1.03627 | up | 9.16918 | 6.89977799 | 9.2254614 | 19.57317 | 7.8015 | 24.50205 |
| ENSSSCG00000006655 | C1orf51 | (4):(-):(108088711-108091789) | 0.00232679 | 1.74457 | up | 3.97161488 | 2.126289546 | 1.594718042 | 7.213432366 | 7.99930878 | 10.56488037 |
| ENSSSCG00000010554 | SCD | (14):(+):(120964206-120980914) | 0.00232679 | 2.04407 | up | 4.59308161 | 6.163485 | 16.47337225 | 30.9843346 | 8.060059 | 73.2537074 |
| ENSSSCG00000002919 | TYROBP | (6):(-):(40711873-40715184) | 0.00232679 | -1.58383 | down | 14.18643 | 61.3743 | 29.8009 | 9.08838 | 6.98453 | 19.07533 |
| ENSSSCG00000011239 | NOVEL GENE | (13):(+):(23827615-23843363) | 0.00232679 | -1.03864 | down | 2.46104 | 5.07459 | 2.64912 | 1.53832 | 1.89562 | 1.52385 |
| ENSSSCG00000009585 | GADD45G | (14):(+):(1421136-1422690) | 0.00232679 | 1.37017 | up | 12.12112 | 16.40014 | 13.220463 | 62.1408 | 16.30995 | 29.45178 |
| ENSSSCG00000017416 | DHX58 | (12):(+):(20937868-20946850) | 0.00232679 | -1.39283 | down | 3.287793 | 16.20373 | 1.816427688 | 2.6601026 | 3.171306 | 2.28305 |
| ENSSSCG00000006379 | CD48 | (4):(+):(97721030-97736619) | 0.00232679 | -1.89325 | down | 1.3026355 | 6.372429 | 3.41205285 | 0.79894 | 1.125007369 | 1.060711631 |
| ENSSSCG00000025499 | FXYD5 | (6):(+):(40131850-40145607) | 0.00232679 | -1.39277 | down | 7.057000204 | 12.9707056 | 9.641365 | 3.509037948 | 3.435151 | 4.354754 |
| ENSSSCG00000010705 | GMFG | (14):(+):(143745490-143752509) | 0.00232679 | -1.92781 | down | 5.61858157 | 48.470403 | 12.07146434 | 3.285279524 | 5.100719 | 9.002856917 |
| ENSSSCG00000013144 | MPEG1 | (2):(+):(11807282-11811580) | 0.00232679 | -1.94543 | down | 1.77923 | 11.9766 | 6.08974 | 1.53475 | 1.39053 | 2.22735 |
| ENSSSCG00000008943 | SLC4A4 | (8):(+):(71840474-72050171) | 0.00232679 | 1.84256 | up | 0.94917 | 1.79117 | 1.04653 | 6.84341 | 5.08035 | 1.6577 |
| ENSSSCG00000028567 | BTNL9 | (2):(-):(159560626-159577201) | 0.00232679 | 1.01928 | up | 10.8807 | 6.25489 | 5.60893 | 17.9457 | 15.2116 | 12.9437 |
| ENSSSCG00000023772 | SPN | (3):(-):(18607803-18612129) | 0.00425221 | -1.3723 | down | 1.12668 | 2.91221 | 1.78514 | 0.691121 | 0.670039 | 0.888515 |
| ENSSSCG00000000025 | PARVG | (5):(+):(2169173-2193456) | 0.00425221 | -1.64738 | down | 1.645638 | 5.72343 | 3.062179 | 0.753536876 | 0.913980162 | 1.662334 |
| ENSSSCG00000017252 | KCNJ2 | (JH118414.1):(-):(92222-93638) | 0.00232679 | 1.36583 | up | 5.719072 | 4.666335344 | 3.76512493 | 12.23949499 | 15.45544922 | 8.7745744 |
| ENSSSCG00000029231 | PigE-108A11.6 | (6):(+):(53902904-53909080) | 0.00605376 | -2.04933 | down | 0.457638879 | 2.821666 | 1.33607382 | 0.502075568 | 0.23848417 | 0.374496741 |
| Pig_newGene_2500 | NOVEL GENE | (18):(-):(7715208-7716580) | 0.00232679 | -1.42834 | down | 5.80949 | 9.68263 | 8.73852 | 1.4016 | 2.8691 | 4.27721 |
| ENSSSCG00000004754 | CHAC1 | (1):(-):(145522853-145525603) | 0.00232679 | 1.39704 | up | 2.60472 | 4.36087 | 0.91541 | 18.59429 | 1.257759918 | 0.903397843 |
| ENSSSCG00000014899 | PRCP | (9):(+):(18898060-18972531) | 0.00232679 | -1.08147 | down | 10.35475624 | 8.839571 | 9.5315748 | 4.440584937 | 3.94059386 | 5.1931694 |
| ENSSSCG00000006736 | CD2 | (4):(-):(113925945-113941749) | 0.00232679 | -1.57838 | down | 1.70197 | 12.49249 | 5.79973 | 1.853211 | 1.676238 | 3.165751 |
| ENSSSCG00000023374 | SRGN | (14):(+):(78211062-78215931) | 0.00232679 | -1.48743 | down | 6.93883318 | 27.470825 | 16.0611 | 5.0392 | 4.823231978 | 8.13778 |
| ENSSSCG00000015595 | ATF3 | (9):(+):(143690556-143703872) | 0.00232679 | -2.52181 | down | 12.05042449 | 14.01615 | 107.29381 | 9.479331445 | 9.093721 | 4.648200514 |
| ENSSSCG00000012805 | SPRY3 | (X):(-):(142640577-142641443) | 0.00232679 | 1.44059 | up | 0 | 0.17045 | 0 | 0.234929 | 0.000824846 | 0.000329737 |
| ENSSSCG00000002471 | ISG12(A) | (7):(+):(122215478-122220545) | 0.00232679 | -1.26251 | down | 85.1744 | 295.017 | 28.1794 | 57.2834 | 58.9627 | 53.9703 |
| ENSSSCG00000014725 | HBB | (9):(-):(5632715-5634344) | 0.00232679 | -1.32842 | down | 264.00254 | 117.748336 | 196.496709 | 163.14848 | 33.984336 | 33.1285 |
| Pig_newGene_844 | NOVEL GENE | (11):(-):(77502387-77533199) | 0.00232679 | 9.11924 | up | 4.778882 | 4.508535593 | 14.16565 | 5.722634687 | 3.210527815 | 8.06508 |
| ENSSSCG00000002279 | GPX2 | (7):(-):(95388198-95392767) | 0.00232679 | 2.30876 | up | 2.157691 | 1.7443624 | 2.121104 | 2.8978 | 7.419587162 | 19.52483 |
| ENSSSCG00000025133 | ITGB2 | (13):(-):(217446028-217475903) | 0.00232679 | -1.67812 | down | 2.83088 | 17.7943 | 10.05979 | 2.544076 | 2.901602 | 4.14303 |
| ENSSSCG00000013888 | JAK3 | (2):(+):(59475336-59495969) | 0.00232679 | -1.35705 | down | 1.298557287 | 2.687869217 | 2.783700972 | 0.751967828 | 0.53187059 | 1.35907772 |
| ENSSSCG00000007198 | ANGPT4 | (17):(+):(39069492-39114089) | 0.00232679 | 1.61994 | up | 0.470071697 | 1.031395359 | 0.9494899 | 3.781024629 | 0.9177297 | 2.834548671 |
| ENSSSCG00000012076 | MX2 | (13):(-):(215028064-215059085) | 0.00232679 | -1.47408 | down | 13.85691383 | 116.101863 | 9.46665954 | 21.04067736 | 14.290596 | 14.856687 |
| ENSSSCG00000009446 | PCDH17 | (11):(+):(32000815-32142625) | 0.00232679 | -1.06064 | down | 4.02685953 | 1.64298942 | 2.85816 | 1.877311957 | 0.875715097 | 1.33545 |
| ENSSSCG00000010044 | IGLC | (14):(-):(52423079-52423532) | 0.00232679 | -3.54031 | down | 33.9552 | 21.8463 | 230.245 | 0.00115023 | 19.329 | 12.3569 |
| ENSSSCG00000015092 | CD3E | (9):(+):(50660810-50675098) | 0.00232679 | -2.27866 | down | 4.13209 | 8.25352 | 7.82203 | 1.06061 | 1.52724 | 1.57673 |
| ENSSSCG00000023124 | ADD1 | (GL896203.1):(+):(206-3526) | 0.00784602 | 1.02573 | up | 4.381367 | 5.049074 | 8.80427 | 13.08122014 | 7.612493 | 16.43181 |
| ENSSSCG00000017087 | GM2A | (16):(-):(77974389-77987649) | 0.00232679 | -1.06184 | down | 4.386359241 | 20.1253 | 8.070664 | 4.94387 | 5.723103 | 4.9406 |
| ENSSSCG00000001081 | SOX4 | (7):(+):(17852990-17854423) | 0.00232679 | -1.64525 | down | 7.56693 | 4.56048 | 7.31261 | 1.964993 | 2.529381 | 3.16431 |
| ENSSSCG00000015559 | NCF2 | (9):(-):(137106911-137137467) | 0.00232679 | -2.16278 | down | 1.26532 | 7.61265 | 2.16565 | 0.660927 | 0.736784 | 1.06861 |
| ENSSSCG00000002554 | PLD4 | (7):(+):(131340863-131347987) | 0.00232679 | -1.39069 | down | 3.13768 | 8.16016 | 7.64273 | 2.35036 | 1.74735 | 3.12589 |
| ENSSSCG00000014326 | KIF20A | (2):(+):(145901081-145910004) | 0.00232679 | -1.58706 | down | 2.389379502 | 2.66024533 | 1.274625754 | 0.919091985 | 0.434930717 | 0.750994966 |
| Pig_newGene_820 | NOVEL GENE | (11):(-):(13842051-13847264) | 0.00232679 |  | down | 0 | 3.29678 | 5.02988 | 0 | 0 | 0 |
| ENSSSCG00000028623 | RARRES1 | (13):(-):(106755175-106799382) | 0.00232679 | -2.47774 | down | 1.32805596 | 7.89988 | 2.95957 | 0.513883 | 0.529683 | 1.144396 |
| ENSSSCG00000023215 | MAOB | (X):(-):(43355507-43474320) | 0.00605376 | 1.06493 | up | 1.755454776 | 2.29061852 | 1.388425354 | 2.454561466 | 6.75112 | 2.163677584 |
| ENSSSCG00000008217 | CD8A | (3):(-):(60852723-60859738) | 0.00232679 | -2.05811 | down | 1.320379022 | 3.840241 | 4.780032726 | 0.806387945 | 0.663802283 | 0.916855 |
| ENSSSCG00000024817 | RASSF2 | (17):(+):(14563017-14588375) | 0.00232679 | -1.23974 | down | 2.23686 | 1.8988 | 2.45189 | 1.140442973 | 0.748771 | 0.900272726 |
| Pig_newGene_4032 | NOVEL GENE | (6):(+):(18527354-18533310) | 0.00425221 | 1.22733 | up | 27.0649 | 22.0923 | 23.2676 | 70.2249 | 63.613 | 35.7325 |
| ENSSSCG00000013594 | NOVEL GENE | (2):(+):(71550710-71573330) | 0.00605376 | -1.07478 | down | 3.846346767 | 2.060146901 | 1.565296447 | 1.022698816 | 1.72208372 | 0.802384598 |
| ENSSSCG00000021964 | PIK3R1 | (16):(-):(50135843-50164238) | 0.00232679 | 1.34553 | up | 4.08801 | 5.92683 | 4.4303 | 14.6314 | 9.12253 | 12.9547 |
| Pig_newGene_2680 | NOVEL GENE | (2):(+):(91705049-91711838) | 0.00232679 | 1.15238 | up | 29.5538 | 24.7489 | 21.2265 | 53.8599 | 68.3719 | 45.655 |
| ENSSSCG00000009216 | SPP1 | (8):(-):(140307938-140315415) | 0.00232679 | -3.42885 | down | 3.28605 | 207.7283 | 16.48262 | 0.493755415 | 17.3985 | 3.23239 |
| ENSSSCG00000009825 | PPTC7 | (14):(-):(33827403-33868403) | 0.00232679 | 1.2186 | up | 21.6891 | 20.8291 | 17.3595 | 45.7183 | 50.0612 | 43.5683 |
